# Comparative Study of the Structural and Vibroelectronic Properties of Porphyrin and Its Derivatives

**DOI:** 10.3390/molecules191220988

**Published:** 2014-12-15

**Authors:** Metin Aydin

**Affiliations:** Department of Chemistry, Faculty of Art and Sciences, Ondokuz Mayıs University, Samsun 55139, Turkey; E-Mail: aydn123@netscape.net; Tel.: +90-362-312-1919 (ext. 5522); Fax: +90-362-457-6081

**Keywords:** Raman, IR, TD-DFT calculation, porphyrin derivatives, PES, IC, ISC

## Abstract

Density functional theory (DFT and time-dependent-DFT (TD-DFT) were employed to investigate the vibroelectronic structural properties of porphyrin and some derivatives: unsubstituted porphyrin (TPyr), *meso*-tetraphenylporphyrin (TPP), *meso*-tetrakis(*p*-sulfonatophenyl)porphyrin (TSPP), protonated-TPyr (H_2_TPyr), deuterated-H_2_TPyr (D_4_TPyr), protonated-TPP (H_2_TPP) and deuterated-H_2_TPP (D_4_TPP), protonated TSPP (H_2_TSPP), deuterated-H_2_TSPP (D_4_TSPP), dicationic TSPP (H_6_TSPP) and deuterated-H_6_TSPP (D_8_TSPP). The possible internal conversion (IC) and intersystem crossing (ISC) processes of these compounds were investigated. Finally, the relaxed ground state potential energy surface (PES) (S_0_), and singlet (S_n_, *n* = 1–24) and triplet (T_n_) excited state PESs of the TSPP molecule were calculated as function of the dihedral angle (C_α_-C_m_-C_ϕ_-C(ph)) rotation. The results of the calculations indicated that while the *meso*-substitutions caused a significant shift in frequencies when the *meso*-carbons within the parent-porphine (or TPyr) are involved in the vibrational motion of molecules; the protonation of the N atoms at the porphine/porphyrin core causes a significant blue shift when the H on the N atoms within the pyrroline are dominantly involved in the vibrational motions. The deuteration of N atoms not only caused a red-shift in the frequencies of the corresponding peaks below 1600 cm^−1^, but also produced new vibrational modes of frequencies in the 2565–2595 cm^−1^ range caused by the N-D bond stretching. Similarly, the deuteration of O atoms within the sulfonato groups (-SO_3_^−^) exhibited a new peak at around 2642 cm^−1^ due to O-D bond stretching. The measured Raman spectrum of the H_2_TSPP is assigned based on the predicted Raman spectra of the compounds studied here and measured Raman spectrum of the TPP (from our previous work). The IR spectrum is assigned based on our calculations and measured IR spectra obtained from the literature. The results of the TD-DFT calculations did not only indicate that the *meso*-substitution and protonation of the porphyrin bring about a significant read shift in the electronic transitions; but also provided a strong evidence for the IC from the Soret band to Q-bands beside possibility of the ISC process; its existence depend on the other excited state process such as much faster vibrational relaxation; the IC and *etc.* The ground state PES curve (S_0_) of the ionic TSPP exhibited two minima at the dihedral angle (C_α_-C_m_-C_ϕ_-C) of about 66° (corresponds to the lowest ground state) and 110° (corresponds to next energetically stable state or the local minima). The energy deference between these two minima is 0.0132 eV (or 106 cm^−1^) and the highest potential energy barrier when undergoing from the lowest ground state to this local state is only 0.0219 eV (177 cm^−1^; which is compatible with the thermal energy (kT) at 298 K is 207.2 cm^−1^.

## 1. Introduction

Porphyrin and its derivatives have attracted considerable attention from both experimentalists and theoreticians for the last few decades and still remain an attractive research subject due to their broad applications in industrial and biomedical fields. The ubiquity of porphyrin derivatives in Nature and their subtle yet crucial chemical and biological functions have motivated scientists to study the unique structure/dynamics characteristics of molecules of this family, and to attempt to mimic their properties, such as high efficiency utilization of solar energy [1,2,3,4,5], and in synthetic molecular analogs, active elements in molecular electronic devices [6,7]. Enormous interest has also arisen in exploiting porphyrin-like molecular systems for the use as therapeutic drugs, photosensitizers in photodynamic therapy of cancer [8], as well their potential applications in the treatment of nonmalignant conditions such as psoriasis, blocked arteries, and pathological and bacterial infections [9], as well as HIV [10]. The biological effects of porphyrins basically depend on their physicochemical properties that essentially cause important changes in their photophysical behavior, for instance, aggregation and axial ligation cause significant changes on their absorption spectra, quantum yield, fluorescence lifetime, and triplet state lifetime [11,12,13]. A inclusive collection entirely devoted to porphyrins may be found in the *Handbook of Porphyrin Science* [14,15].

Very few porphyrins are known to form J-aggregates, the main requisite being the zwitterionic character with the protonation of the pyrrole nitrogen within the macrocycle. It has also been reported that these aggregates may be promoted by interaction with proteins [16,17] and surfactants [18]. The aggregation of the anionic porphyrin *meso*-tetrakis(*p*-sulfonatophenyl)porphyrin sodium salt (H_2_TSPP) has been studied extensively. In aqueous solutions, at neutral pH, the electronic absorption spectrum of *meso*-tetrakis(*p*-sulfonatophenyl)porphyrin (H_2_TSPP) is usual of free base porphyrins (D_2h_ symmetry) and is characterized by an intense Soret band at around 420 nm and four Q bands in the region of 500–700 nm. In very acidic medium, H_2_TSPP forms highly ordered molecular J and H aggregates [9,19,20,21]. The absorption spectrum of aggregated H_2_TSPP in acidic medium exhibits two new bands at 490 and 707 nm [21].

Quantum chemical calculations are expected to play a crucial role in promoting the ends mentioned above since the ideal approach for acquisition of intrinsic structural information of molecules, especially those of large sizes such as the porphyrinoids, would be through theoretical calculations, in which structural information would not be influenced by solvent interaction or crystal packing effects—though correction for such phenomena might be accomplished through appropriate modeling; moreover, quantum chemical calculations would be inherently free of the arbitrariness associated with deciphering structure characteristics from spectroscopic observations. Recent advances in computing facilities and the development of sophisticated computation programs, with increasingly efficient algorithms, especially the fundamental improvements in the treatment of electron correlation based on density-functional theory (DFT) [22], have also combined to allow quantum chemical methods to routinely handle molecular systems containing hundreds of atoms. Density functional theory (DFT) is also one of the most important techniques used by theoreticians to provide deep insight into spectroscopic and structural properties, even for complex molecular systems, especially those of large sizes such as the porphyrinoids [23,24,25,26,27,28].

In the present work, in order to investigate the effect of *meso*-substitution groups on the geometric and vibroelectronic properties of the parent-porphine or porphyrin macrocycle, density functional theory (DFT) and time-dependent-DFT (TD-DFT) were employed to investigate the vibroelectronic structural properties of porphyrin and derivatives: unsubstituted porphyrin (TPyr), *meso*-tetraphenylporphyrin (TPP), *meso*-tetrakis(*p*-sulfonatophenyl)porphyrin (TSPP), protonated-TPyr (H_2_TPyr), deuterated-H_2_TPyr (D_4_TPyr), protonated-TPP (H_2_TPP or dicationic TPP) and deuterated-H_2_TPP (D_4_TPP), protonated TSPP (H_2_TSPP or dianionic TSPP), deuterated-H_2_TSPP (D_4_TSPP), dicationic TSPP (H_6_TSPP) and deuterated-H_6_TSPP (D_8_TSPP). The possible internal conversion (IC) and intersystem crossing (ISC) processes for the porphyrin and derivatives were also investigated by using the TD-DFT technique. Furthermore, the relaxed potential energy surface scan were employed to study the minimum potential energy pathways for the ground and excited states of the TSPP molecule as a function of rotation Cm-C_ϕ_ bond (or dihedral angle (C_α_-C_m_-C_ϕ_-C(ph)). We, further, ascertain that the experimental Raman bands of the H_2_TSPP molecule can most appropriately be described in terms of the concerted vibrations of local structural units such as the pyrrole/pyrroline rings, the sulfonatophenyl groups, and their combinations, rather than as vibrations of isolated chemical bonds, as has been the common practice. The calculated IR spectra of the H_2_TSPP were assigned by comparison with the calculated IR spectra of other porphyrin derivatives studied here and the experimentally measured IR spectra obtained from the literature.

## 2. Results and Discussion

### 2.1. Structures of Porphyrin and Its Derivatives

**Figure 1:**
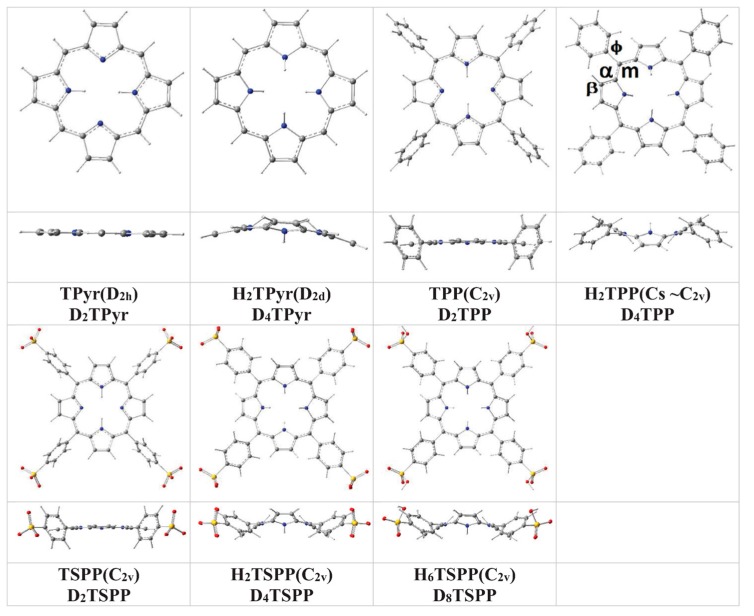
Molecular structures of unsubstituted porphyrin (TPyr), *meso*-tetraphenylporphyrin (TPP), and anionic meso-tetrakis(*p*-sulfonatophenyl)porphyrin (TSPP) as well as their protonated structures (H_2_TPyr, H_2_TPP, H_2_TSPP and H_6_TSPP) in water at the B3LYP/6-311G(d,p) level of the DFT.

In brief, the predicted ground state geometry and selected bond angles and dihedral (torsional) angles of the porphyrin and its derivatives in water, used as a solvent, at B3LYP/6-311G(d,p) level of the DFT are provided in Figure 1 and Table 1. the results of the calculations indicated that while the *meso*-substitution of porphyrin with tetraphenyl or tetrasulfonatophenyl cause slightly out-of-plane distortion from the planar structure of the macrocycle (within 3° to 5°) for TPP and TSPP; the protonation of the porphyrin core leads to a significant distortion from its planarity within 10° to 20° for H_2_TPyr, H_2_TPP, H_2_TSPP and H_6_TSPP. Furthermore, with respect to the average plane of porphryin to macrocycle as seen in Figure 1 and Table 1, the peripheral phenyl and sulfonatophenyl substituents are oriented at a tilt angle of about 72° for the TPP and TSPP (unprotonated structures) and about 48° for the H_2_TPP, H_2_TSPP and H_6_TSPP (protonated structures).

**Table 1:** Selected dihedral angles (D) and bond angles (A) of free-base porphyrin (TPyr), *meso*-tetraphenylporphyrin (TPP), and the anionic meso-tetrakis(*p*-sulfonatophenyl)porphyrin (TSPP) as well as their protonated structures (H_2_TPyr, H_2_TPP, H_2_TSPP and H_6_TSPP). The calculations were done in water at the B3LYP/6-311G(d,p) level of the DFT.

	TPyr	TPP	TSPP	H_2_TPyr	H_2_TPP	H_2_TSPP	H_6_TSPP
D(C_β_, C_α_, C_m_, C_ϕ_)	N.A	3.6	4.0	N.A	19.4	19.6	19.0
D(C_β_, C_α_, C_m_, C_α__′_)	180.0	−176.5	−176.4	−169.5	−166.6	−160.5	−161.6
D(C_α_, C_m_, C_α’_, N)	0.0	2.3	2.4	10.3	20.6	20.7	20.0
D(C_α_, C_m_, C_ϕ_, C)	N.A.	72.1	71.0	N.A	47.8	47.2	49.6
D(C_α_, C_m_, C_α__′_, C_β__′_)	180.0	−177.2	−176.9	−169.5	−160.6	−160.5	−160.8
D(C_m_, C_α__′_, C_β__′_, C_β__″_)	180.0	179.8	179.7	−178.8	−176.4	−174.4	−176.9
D(C_α__′_, C_β__′_, C_β__″_, C_α__″_)	0.0	0.0	0.0	0.0	0.0	0.0	0.0
D(C_β__′_, C_β__″_, C_α__″_, C_m__′_)	180.0	−179.8	−179.7	178.8	176.4	176.4	176.9
D(C_β__″_, C_α__″_, C_m__′_, C_ϕ__′_)	N.A	−2.6	−2.6	N.A	−19.4	−19.4	−18.9
D(C_α__″_, C_m__′_, C_ϕ__′_, C)	N.A	−72.1	−70.9	N.A	−47.8	−47.5	−49.9
D(C_α__′_, N, C_α__″_, C_β__″_)	0.0	0.4	0.4	2.2	4.2	4.2	4.1
A(C_β_, C_α_, C_m_)	127.9	123.0	123.1	127.7	128.0	128.0	128.0
A(C_α_, C_m_, C_ϕ_)	N.A	118.2	118.2	N.A	118.3	118.3	118.2
A(C_ϕ_, C_m_, C_α__′_)	N.A	116.6	116.5	N.A	118.3	118.4	118.4
A(C_α_, C_m_, C_α__′_)	127.0	125.2	125.3	127.4	123.4	123.4	123.9
A(C_m_, C_α__′_, C_β__′_)	123.4	126.9	126.9	127.7	128.0	128.0	127.9
A(N, C_α_, C_m_)	125.6	126.3	126.2	125.5	125.4	125.3	125.2
A(C_m_, C_α__′_, N)	125.7	126.6	126.6	125.5	125.4	125.3	125.3

The calculated bond lengths are in agreement with X-ray measurements within *ca.* ±0.01 Å. As result, the calculations indicated clearly indicated that the protonation of the porphyrin core does not only destroy planarity of the macrocycle, but also, has an effect on the tilt angle of the phenyl and *p*-sulfonatophenyl substitution groups.

### 2.2. Raman Spectra of Porphyrin and Derivatives

**Figure 2:**
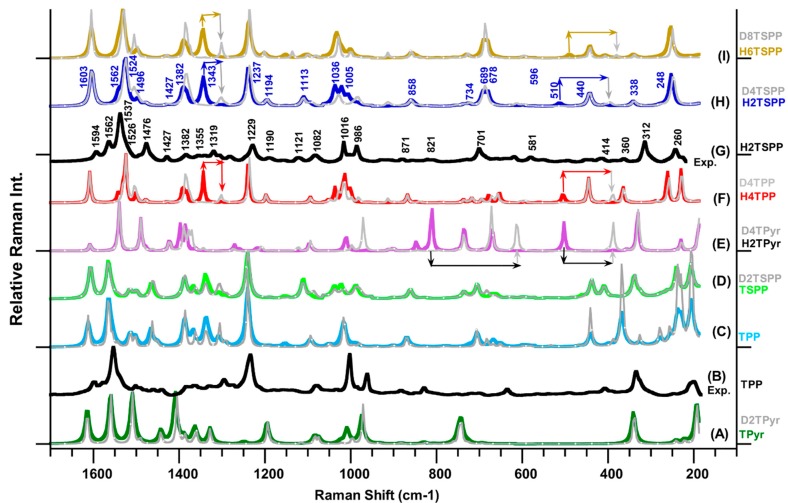
Calculated Raman spectra of porphyrin derivatives in water (used as solvent in the calculations) at the B3LYP/6-311G(d,p) level of the DFT: (A) free-base porphyrin (TPyr) and deuterated-TPyr (D_2_TPyr); (C) *meso*-tetraphenylporphyrin (TPP) and (D_2_TPP); (D) anionic *meso*-tetrakis(*p*-sulfonatophenyl)porphyrin (TSPP) and deuterated-TSPP (D_2_TSPP), and (E) protonated-TPyr (H_2_TPyr) and deuterated-H_2_TPyr (D_4_TPyr); (F) protonated-TPP (H_2_TPP) and deuterated-H_2_TPP (D_4_TPP); (H) protonated TSPP (H_2_TSPP) and deuterated-H_2_TSPP (D_4_TSPP); and (I) dicationic TSPP (H_6_TSPP) and deuterated-H_6_TSPP (D_8_TSPP). The measured Raman spectra of: (B) TPP (taken from ref. [23]) and (G) H_2_TSPP. It should be noted that the plot Raman spectra in the gray color belong to the deuterated molecules, and the line arrows show the frequency shift in the deuterated molecule.

**Figure 3:**
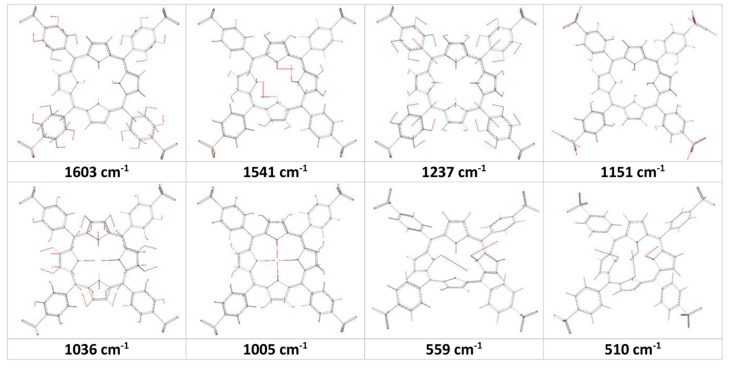
Calculated molecular motions for some vibrational bands of the H_2_TSPP.

Figure 2 provides the measured Raman spectra of the TPP (Figure 2B, from our previous work [23] and H_2_TSPP (Figure 2G), which reveal many Raman peaks with strong and medium intensity as well as many weak or very weak features dispersed through the spectrum. When comparing the observed Raman spectrum of the H_2_TSPP with the TPP, the Raman patterns in spectra of both molecules are very similar, but some of the Raman peaks positions are significantly shifted in frequency. For instance, while the most intense peak at 1564 cm^−1^ and relatively weak peaks at 1595, 1577, 1438, 1327, 1234 and 334 cm^−1^ in the spectrum of TPP are red-shifted to 1537 cm^−1^ (the strongest one), 1494, 1562, 1427, 1339, 1229 and 312 cm^−1^, respectively. The peaks at 1002, 962, 334 and 201 cm^−1^ are respectively blue-shifted to 1014, 983, 314 and 236 cm^−1^ in the spectrum of H_2_TSPP. Also, the Raman bands at 1476 and 701 cm^−1^ Raman spectrum of the H_2_TSPP are significantly enhanced reference to corresponding peaks in the TPP spectrum. Figure 2 provide the calculated Raman spectra of the TPyr/D_2_TPyr, H_2_TPyr/ D_4_TPyr, TPP/D_2_TPP, H_2_TPP/(D_4_TPP), TSPP/D_2_TSPP, H_2_TSPP/D_4_TSPP, and H_6_TSPP/D_8_TSPP in water (used as a solvent), with the observed Raman spectra of the TPP and H_2_TSPP for comparison. (It is worthy to note that where D indicates that deuterium atoms covalently bound to the N atoms at porphyrin core; for dicationic D_6_TSPP, four of eight D atoms covalently bound to O atoms within sulfonato group, -SO_3_^−^, other four covalently bound to the N atoms at the core). While only the data for the Raman features of TPP, TSPP and H_2_TSPP are presented in Table 2; the observed Raman spectrum of the H_2_TSPP is assigned in connection with the calculated Raman spectra of the compounds studied here and observed Raman spectrum of TPP (from our previous work [23]). Our conclusion may be summarized as follows: 

(1) the Raman peak at 1593 cm^−1^: as seen in Figure 2, the calculations produced an active Raman band at about 1600 cm^−1^ for all of the porphyrin derivatives. However, the nuclear motion of the molecules showed that this calculated Raman band is due to C-C bond stretching ν(C-C) within phenyl rings and rocking of their H ρ(CH), no any contribution comes from macrocycle and sulfonato groups (-SO_3_^−^). The spectra of the free base porphyrin (TPyr) and protonated TPyr (H_2_TPyr) produced a peak around 1600 cm^−1^, which results from asymmetric stretching of the C_α_-C_m_-C_α_ (ν_a_(C-C_m_-C)) and bending deformation of the C-N(H)-C/C-N-C bonds, θ(C-N(H)-C/C-N-C), and rocking of H on C_m_ atoms (ρ(C_m_H)), as seen in Figure 3. Therefore, the vibrational motion of the phenyl substitution is responsible for the observed Raman band at 1593 cm^−1^ in the observed spectrum of protonated TSPP (H_2_TSPP). Furthermore, in our previous work [23], the measured and calculated Raman spectrum of the TPP revealed the same result and also these results are shown in Table 2 and Figure 2.

(2) The observed peak at 1563 cm^−1^: while the measured Raman spectrum of the H_2_TSPP displayed a relatively weak bands at 1563 cm^−1^, its calculated Raman spectrum displayed only an extremely very weak peak at 1568 cm^−1^ in this region (as a result of C-C bond stretching within phenyl rings and rocking of their H, accompanied by relatively weak asymmetric stretching of C_α_-C_m_-C_β_ bond stretching, no any contribution comes from sulfonato groups). When we examined this peak caused by the same vibrational motion for the TPP, TSPP, H_2_TPP and H_6_TSPP, their calculated Raman spectra again exhibited an extremely very weak peak at 1588, 1574, 1585 and 1578 cm^−1^, respectively. Actually, the calculated spectrum of the TSPP produced the most intense Raman band at 1,564 cm^−1^, but its vibrational motions indicated that this mode of frequency shifted to 1524 cm^−1^ in the spectrum of H_2_TSPP. This problem is not only observed for H_2_TSPP, but also, we found the same problem for the TPP (seen Figure 2 and Table 2). Therefore, we believe that the DFT calculations might underestimate the intensity of this peak.

(3) The observed Raman spectra of TPP and H_2_TSPP exhibited a strong band at 1553 cm^−1^ (with a shoulder *ca.* at 1540 cm^−1^) and at 1537 cm^−1^ (with a shoulder at *ca.* 1528 cm^−1^), respectively. Their calculated spectra displayed the strongest Raman band at 1564 and 1524 cm^−1^, respectively, as a results of the same vibrational motion: C_β_-C_β_ bond stretching, ν(C_β_-C_β_), symmetric stretching of C_α_-C_m_-C_α_ bonds, ν_s_(C_α_-C_m_-C_α_), which lead to bending deformation of the C-N-C bonds, θ(C-N(H)-C). The observed shoulders at 1540 cm^−1^ (TPP) and at 1528 cm^−1^ (H_2_TSPP) correspond to the calculated peaks at 1559/1540 cm^−1^ (in the TPP/H_2_TSPP and caused by ν_s_(C_α_-C_m_-C_α_)/ρ(C-N(H)-C)/ρ(NH)) and 1555/1529 cm^−1^ (in the TPP/H_2_TSPP, owing to ν(C_β_-C_β_)/ρ(C_β_H) and relatively weak θ(C-N(H)-C)). Both of the experimental and calculated Raman spectra showed that the strongest band in the TPP spectrum is significantly red shifted in the H_2_TSPP spectrum. This large red-shift in this peak position (about exp. 14 cm^−1^ and *ca.* 40 cm^−1^) is mainly caused by the out-of-plane distortion of the macrocycle as a result of protonation of the N atoms at porphyrin core, not due to the substitution effect, which is responsible from the observed Raman band at 1563 cm^−1^ in the spectrum of H_2_TSPP. Also, the frequency shift of this peak in the calculated spectra of the TPyr, TPP and TSPP is found in the calculated spectrum of the H_2_TPyr, H_2_TPP and H_6_TSPP. 

**Table 2:** Observed and calculated Raman active modes of frequencies (in cm^−1^) of the H_2_TSPP (C_2v_) with the TPP (C_2v_ point group) and TSPP (C_2v_) for comparison. The calculations were carried out in water used as solvent at B3LYP/6-311G(d,p) level of the DFT. Where ∆ν_sc_ stands for the scaled vibrational frequencies (∆ν_sc._ = 0.96∆ν_calc_ +40) and S_R_ and I_R_ indicate the calculated Raman scattering activity and intesity, respectively; **∆**ν_exp_ and I_R/exp_ stand for the observed Raman frequency and Intensity, respectively. The experimental values of Raman spectrum of the TPP are taken from our previous work [23].

TPP						TSPP					H_2_TSPP					
	∆ν_sc._	S_R_	I_R_	$∆{\text{ν}}_{\text{exp}}^{\text{Ref.23}}$	${\text{I}}_{\text{R/ exp}}^{\text{Ref.23}}$	H2	∆ν_sc._	S_R_	I_R_	H4	∆ν_sc._	S_R_	I_R_	∆ν_exp_	I_R/exp_	
A2	1612	25	24	1595	29	A2	1607	44	42	A2	1603	45	40	1593	21	C-C bond stretching within phenyl rings and rocking of their H, no any contribution comes from macrocycle and sulfonato groups (-${\text{SO}}_{3}^{-}$).
A1	1612	29	27			A1	1607	53	50	A1	1603	55	49			
A1	1588	<1	<1	1577	27	A1	1574	1	1	A1	1568	2	1	1563	42	C-C bond stretching within phenyl rings and rocking of their H, accompanied by relatively weak asymmetric stretching of C_α_-C_m_-C_β_ bond stretching, no any contribution comes from sulfonato groups.
A1	1564	100	100	1553 1540	100 40	A1	1564	100	100	A1	1524	100	100	1537	100	C_β_-C_β_ bond stretching, ν(C_β_-C_β_), symmetric stretching of C_α_-C_m_-C_α_ bonds, ν_s_(C_α_-C_m_-C_α_) that leads to bending deformation of the C-N-C bonds, θ(C-N(H)-C).
A2	1559	8	8			A2	1558	9	9	A2	1540	29	28	1528	42	ν_s_( C_α_-C_m_-C_α_)/rocking of C-N(H)-C and H on N atoms, ρ(C-N(H)-C)/ρ(NH)
A1	1555	15	15			A1	1554	8	8	A1	1529	26	25			Asymmetric stretching of C_α_-C_m_-C_α_) bonds ν_a_(C_α_-C_m_-C_α_)/θ(C-N(H)-C).
A1	1514	24	25	1502	21	A1	1515	21	23	A1	1477	5	5	1476	38	ν(C_β_-C_β_) and rocking of the H on C atoms within macrocycle (not on the phenyl groups), ρ(C_β_H), and relatively weak θ(C-N(H)-C)
A2	1502	14	15	1491	16	A2	1499	14	15	A1	1496	7	7	1489	15	ρ(CH within phenyl groups only)
A1	1501	4	4			A1	1498	3	4	A2	1495	8	8			
A2	1466	31	36	1461	13	A2	1464	37	44							ν(C_m_-C_α_)/ρ(C_β_H), and relatively weak ν_a_(C-N(H)-C)
A1	1454	3	3	1438	12	A1	1454	2	2	A1	1426	3	3	1428	11	ν_s_(C_α_-C_m_-C_α_)/ρ(C-N(H)-C)/ρ(CH)
A2	1387	46	61	1378	20	A2	1387	44	58	A2	1391	31	38	1384	15	ν_a_(C_β_-C_α_-N(H))/ρ(C_β_H-C_β_H)
A1	1367	22	30			A1	1367	22	29	A1	1382	20	25	1354	14	ν(C_β_-C_α_)/θ(C-C_m_-C)/θ(C-N(H)-C), which leading to macrocycle getting a square shape)
A2	1339	19	26	1327	20	A2	1338	42	60	A2	1343	52	70	1340	14	ν(C_ϕ_-C_m_)/ρ(C_β_H)/ρ(NH), and relatively weak ν_a_(C-N(H)-C)
A2	1335	26	38			A2	1329	13	19					1319	22	
A1	1306	13	19			A1	1305	7	10	A1	1321	1	1	1304	14	ν_a_(C-C-C) within phenyl groups/θ(C-N(H)-C)/ρ(CH).
A1	1291	4	7			A1	1290	4	6	A1	1300	1	2	1283	12	
A1	1239	75	127	1234	84	A1	1239	77	131	A1	1237	60	97	1229	34	ν(C_ϕ_-C_m_) (primarily)/ν_s_(C-N(H)-C)/ρ(CH)/ν(C_β_-C_β_) (relatively weak)
A2	1189	0.	0.			A2	1188	1	1	A2	1194	3	6	1190	8	ρ(CH) within phenyl groups.
A1	1189	1	2			A1	1189	1	1	A1	1194	5	9			
A2	1152	4	8	1137	10	A2	1153	3	5							ρ(NH)/ρ(C_β_H) and relatively weak structural deformation
						A2	1146	<1	<1	A2	1151	<1	<1	1122	9	ν_a_(O-S-O) within sulfonato groups
A1	1097	1	2	1080	21	A1	1109	19	42	A1	1108	9	18	1082	14	ν(S-O)/θ(C-C(S)-C) within sulfonato groups.
A1	1091	5	11			A1	1092	3	6	A1	1093	2	4			ρ(C_β_H)
A1	1048	2	6			A1	1036	1	3	A1	1033	<1	1			θ(C-C-C) within the phenyl groups
A2	1013	6	15			A2	1037	1	4	A2	1035	1	3			
A1	1020	4	10	1002	85	A1	1020	2	5	A1	1036	3	7	1016	40	Expansion of the pyrrole/pyrroline groups along N(H)…N(H) direction due to ν(C_α_-C_β_), leading to macrocycle getting rectangular shape instead of square shape.
A1	983	2	4	962	44	A1	986	2	5	A1	1005	2	5	1002	15	Expansion of the pyrrole/pyrroline groups along N(H)…N(H) direction in the same phase like macrocycle getting square shape or similar to breathing of the macrocycle
A2	990	1	2			A2	988	1	2	A2	992	0.	1			Out of plane wagging of the H on the phenyl rings, w(CH)
A1	899	1	3			A1	899	1	3	A1	904	<1	1	986	32	Bending deformation inside entire molecule.
A1	868	3	13			A1	859	4	14	A1	858	2	8	879	6	w(CH on the macrocycle and phenyl rings)
A1	815	<1	<1			A1	814	<1	<1	A1	820	<1	1	821	5	
A1	768	<1	<1			A1	749	<1	1	A1	751	1	2			Out of plane bending deformation of whole molecule including w(CH/NH)
A1	727	<1	<1			A1	734	3	15	A1	734	1	5	728	5	ν(S-O) and expansion of the phenyl rings along S…C_m_ direction including w(CH/NH)
										A2	689	5	31	701	27	Out of plane twisting of the macrocycle
A1	665	2	12	636	13	A1	669	1.33	9	A1	678	5	33			
A1	584	<1	2			A1	589	<1	1	A1	596	<1	3	580	10	w(NH and CH on the macrocycle and phenyl rings) and wagging of the macrocycle.
A1	531	0.	3			A1	574	0.	1	A1	566	0.	1	548	5	Wagging of entire molecule
										A1	510	0.	2	494	3	w(NH)
A2	468	<1	1			A2	457	1	13	A2	468	<1	1			In-plane wagging of macrocycle and translational motion of phenyl rings.
A2	437	2	33	408	15	A2	435	2	25	A2	440	2	25	439	5	Out of plane bending of the phenyl rings.
						A1	407	2	47	A1	404	<1	6	414	8	Breathing macrocycle and translational motion of phenyl rings in opposite phase.
A1	365	6	160	334	50	A1	336	3	111	A1	338	1	24	314	41	Breathing of whole molecule.
A2	322	<1	17											363	7	Out of plane wagging of macrocycle.
A1	235	2	127	201	29	A1	237	2	152	A1	248	2	144	242	26	Out of plane wagging of macrocycle.
A1	252	<1	28			A1	257	<1	18	A1	252	<1	12			Out of plane wagging of phenyl rings and relatively weak out of plane wagging macrocycle.

(4) The calculated Raman spectra of the TPP, H_2_TPP, TSPP, H_2_TSPP and H_6_TSPP revealed a relatively strong peak around 1238 cm^−1^, arise from the ν(C_ϕ_-C_m_) (predominantly), ν_s_(C-N(H)-C), ρ(CH) and ν(C_β_-C_β_) (relatively weak). This vibrational mode of frequency is assigned to the observed Raman bands at 1229 cm^−1^ (H_2_TSPP) and 1234 cm^−1^ (TPP). It is clear that this mode is mainly due to bond stretching between the *meso*-position C atom (C_m_ within macrocycle) and the adjacent C atom (within the *meso*-substituted phenyl or *p*-sulfonatophenyl groups). For the unsubstituted free base porphyrin molecules, this peak is red-shifted to 1194 cm^−1^ and 1217 cm^−1^ in the calculated spectra of the TPyr and H_2_TPyr, respectively, which mainly result from rocking of the H atom (covalently bound to *meso*-carbon atom), ρ(C_m_H), including vibrational bond stretching within the macrocycle (see Figure 3). The question here is that why this peak is not shifted in the *meso*-substituted phenyl/sulfonatophenyl porphyrin molecules, but it is shifted in the TPyr and H_2_TPyr? This may be explained by the existence of the electrostatic repulsion interactions between the H atoms on the C_m_ atoms and H atoms on the β-C atoms (C_β_) within the pyrrole/pyrroline rings in the case of the TPyr and H_2_TPyr. For instance, this frequency shift in the TPyr (1194 cm^−1^) is larger than in the H_2_TPyr (214 cm^−1^) due to the out of plane distortion from planarity of the protonated porphyrin (H_2_TPyr, see Figure 1) that leads to decreasing electrostatic repulsion interaction between the H atoms on the C_β_ and C_m_ atoms resulting from increasing distance between them.

In the case of *meso*-phenyl/sulfonatophenyl-substituted porphyrin molecules, this electrostatic repulsion interaction between the H atoms on the C_β_ atoms and H atoms on the *meso*-phenyl substituent lead to rotation of the *meso*-phenyl/sulfonatophenyl groups about C_m_-C_ϕ_ bond, up to the tilt angle of 71° for unprotonated structures and about 48° for the protonated porphyrin molecules (relative to macrocycle, see Figure 1 and Table 1). Therefore, due to decreasing in these electrostatic repulsion interactions, the calculations did not produced a significant frequency shift in this peak position (~1238 cm^−1^) in the Raman spectra of the substituted porphyrin molecules.

(5) In the range of 1050–950 cm^−1^, there are two well-known Raman band that are influenced by the protonation and deuteration of the macrocycle. While the observed Raman spectrum of the TPP (excited at 488 nm) exhibited two bands at 1002 and 962 cm^−1^, which are respectively blue shifted to 1016 and 986 cm^−1^ in the H_2_TSPP spectrum (excited at 514 nm). These large shift in the peak positions may be due to protonation of the porphyrin core, which leads to saddle-type distortions of the porphyrin core (in the other words, leading to increasing the degree of the freedom of the rocking of the N-H bonds as a result of decreasing electrostatic repulsion between these H atoms). Furthermore, the Gaussview animation software showed that the band at 1036 cm^−1^ (H_4_TSPP) is due to expansion of the pyrrole/pyrroline groups along N(H)…N(H) direction in opposite direction (Figure 3), as a result of the ν(C_α_-C_β_), leading to macrocycle getting rectangular shape instead of square shape, and the bands at 1005 cm^−1^ (H_4_TSPP) is due to expansion of the pyrrole/pyrroline groups along N(H)…N(H) direction in the same phase like macrocycle getting square shape or similar to breathing of the macrocycle or the breathing of the pyrrole rings as assigned by Rich and McHale [29].

(6) Two other important peaks in low frequency region are predicted at 248 and 338 cm^−1^ for H_2_TSPP and 235 and 365 cm^−1^ for TPP are due to out of plane twisting of the macrocycle only and breathing of entire molecule, respectively, which are consistent with their experimental values (242 and 338 cm^−1^ for the H_2_TSPP; 235 and 334 cm^−1^ for the TPP). These observed and calculated Raman bands of TPP are red and blue shifted as a result of the protonation of the macrocycle, not due to sulfonato-substituted groups. The rest of the assignments of the observed Raman bands are given in Table 2.

#### Isotopic Effect on the Raman Spectrum

The results of the calculations showed that the deuteration of the N atoms at the porphyrin core also produce a significant red shift in their frequencies. There is a strong evidence on this observation comes from the polarized resonance Raman scattering (RRS) spectra of the aggregated H_2_TSPP (protonated TSPP) and D_2_TSPP (deuterated TSPP) by Rich and McHale [29]. The authors reported that the RRS spectra of the aggregated H_2_TSPP and D_2_TSPP (excited at 488 nm) exhibits frequency shifts of some of the well-known Raman modes besides changes in the relative intensities of the Raman modes upon deuteration. They found the most notable frequency shifts observed for the Raman bands at 983 cm^−1^ and 1013 cm^−1^ in the Raman spectrum of protonated TSPP (H_2_TSPP) aggregate that shift to 957 and 1004 cm^−1^ in the spectrum of aggregated D_2_TSPP. They also concluded that these two modes are pyrrole breathing modes and thus the red shifts can be attributed to the substitution of deuterium ions with the labile protons in the porphyrin core [29]. Our measured Raman spectra of the TPP and H_2_TSPP, and protonated and deuterated spectra of aggregated H_2_TSPP and D_4_TSPP clearly showed that while the protonation of the porphyrin core leads to blue shift in the frequency, the deuteration leads to red shift in the spectral position of these two Raman bands.

By comparing the spectral positions of these two peaks in the calculated Raman spectra of protonated and deuterated porphyrin core with their corresponding unprotonated ones (see Table 3), we can see that while the protonation of the free base and meso-substituted porphyrin core caused a blue shift in frequency of these two bands, the deuteration caused a red shift. For instance, while these two Raman band at 1020 and 985 cm^−1^ in the spectrum of TSPP are blue shifted to 1036 and 1005 cm^−1^ in the protonated TSPP (H_2_TSPP), these peaks are red shifted to 1012 and 980 cm^−1^ in the spectrum of deuterated TSPP (D_2_TSPP). When the four N atoms at the core are deuterated (D_4_TSPP), then, these two peaks are shifted from 1036 and 1005 cm^−1^ (in the H_4_TSPP) to 1026 and 983 cm^−1^ in the D_4_TSPP.

**Table 3:** The predicted Raman active bands of frequencies (for the protonated (see Table 2 for TPP and H_2_TSPP) and deuterated (Ref. [29]) porphyrin derivatives) that experimentally exhibited significant frequency shift in the range of 1040–950 cm^−1^.

**TPyr**	**H_2_TPyr**	**TPP**	**Exp.**	**H_2_TPP**	**TSPP**	**H_2_TSPP**	**Exp.**	**H_6_TSPP**
1007	1013	1020	1002	1036	1020	1036	1016	1036
972	1010	983	962	1002	985	1005	983	1001
**D_2_TPyr**	**D_4_TPyr**	**D_2_TPP**		**D_4_TPP**	**D_2_TSPP**	**D_4_TSPP**	**Exp. [29]**	**D_6_TSPP**
996	995			1026	1012	1026	1004	1026
968	968			980	977	983	957	979

The effect of the deuteration of the N atoms on the frequency shift in the rest of intense Raman bands in the calculated spectrum are less than 5 cm^−1^, which is consistent with the experimental observation [29], but there are many predicted Raman bands with weak intensities which exhibited large shift in the frequency (see Figure 2). The other *meso*-substituted and free base porphyrin derivatives showed the similar results, which are in accordance with experimental observations as discussed above. Furthermore, the theoretical calculations indicated that the *meso*-substituted groups do not any significant effect on the spectral position of these two Raman bands. The blue shift of these two peaks for the protonation of the porphyrin core is not so surprising when taking into account of the electrostatic repulsion effect between H atoms (covalent bonded to N atoms) at the porphyrin core. This effect can be minimized by out of plane distortion (or deviation from the planarity) of the macrocycle as discussed above. The red shift also can be expected due to isotopic effect since the vibrational frequency is inversely proportional to the square root of mass of atoms that contribute to the vibrational mode. Furthermore, the deuteration of the porphyrin core and one of oxygen atoms within the sulfonato (SO_3_D) groups exhibited new Raman peaks in the range of 2630–2720 cm^−1^.

### 2.3. IR Spectra of Porphyrin and Derivatives

**Figure 4:**
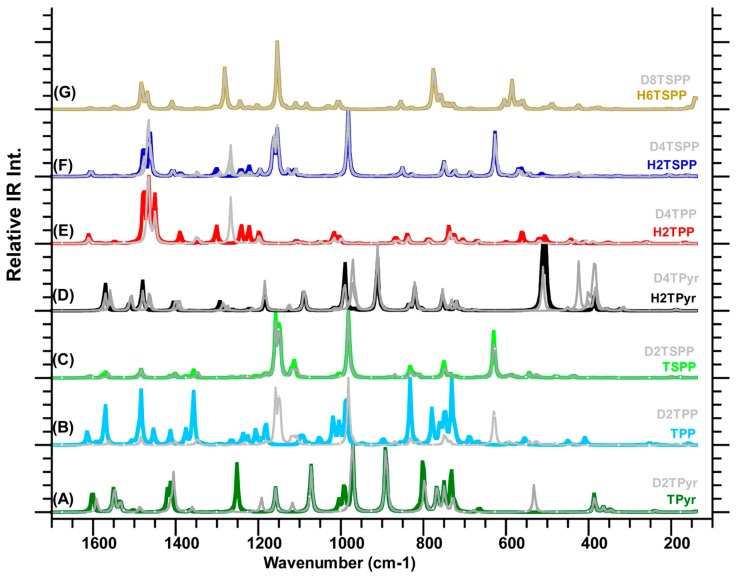
Calculated IR spectra of porphyrin derivatives calculated in water used as solvent at the B3LYP/6-311G(d,p) level of the DFT: (A) free-base porphyrin (TPyr) and deuterated-TPyr (D_2_TPyr); (B) *meso*-tetraphenylporphyrin (TPP) and (D_2_TPP); (C) anionic *meso*-tetrakis(*p*-sulfonatophenyl)porphyrin (TSPP) and deuterated-TSPP (D_2_TSPP), and (D) protonated-TPyr (H_2_TPyr) and deuterated-H_2_TPyr (D_4_TPyr); (E) protonated-TPP (H_2_TPP) and deuterated-H_2_TPP (D_4_TPP); (F) protonated TSPP (H_2_TSPP) and deuterated-H_2_TSPP (D_4_TSPP); and (G) dicationic TSPP (H_6_TSPP) and deuterated-H_6_TSPP (D_8_TSPP). It should be noted that the plot IR spectra in the gray color belong to the deuterated molecules.

We also calculated the IR spectra of TPyr, TPP, TSPP, H_2_TPyr, H_2_TPP, H_2_TSPP and H_6_TSPP as well as their deuterated structures (D_2_TPyr, D_2_TPP, D_2_TSPP, D_4_TPyr, D_4_TPP, D_4_TSPP and D_6_TSPP, where D_2_ and D_4_ indicate the number of D atoms at porphyrin core and D_6_ stands for the deuteration of the porphyrin core and sulfonato groups) at the same level of the DFT. Their spectra exhibited many IR features with medium and relatively weak intense in addition intense IR bands, which are dispersed throughout the full spectral range as seen in Figure 4. The calculations coupled with the animated motions indicated that these calculated IR vibrational modes are principally associated with: (a) symmetric and asymmetric skeletal deformations of the macrocycle and phenyl rings; (b) out-of-plane deformation of the macrocycle and the phenyl rings; and (c) rocking and wagging of the CH and NH bonds. The assignments of selected IR features for these molecules are provided in Table 4. In order to test the reliability of the calculated IR spectra of the molecules investigated here, we also compared their IR peaks in the calculated spectra of the TPP and H_2_TSPP (protonated TSPP) with its experimentally measured IR spectra of TPP [30] and H_2_TSPP [31]. As seen in Table 4, the calculated and measured IR frequencies are well correlated, which indicates that the calculated IR spectra of the molecules investigated in this work are reasonable. We should point out that two types of scaling factors used for the IR spectrum of TPP: one (0.96 × ω_cal_ + 40 in cm^−1^) that we used through paper for the Raman and IR spectra discussed here, but a scaling factor of 0.976 provided best fit to experimentally measured IR data (not for Raman) of TPP only. Only the latter one is given in Table 4.

The key conclusions on the calculated IR spectra as following: (1) when carefully examination the predicted IR features for the H_2_TSPP in connection with the IR spectra of the TPyr/H_2_TPyr, TPP/H_2_TPP and TSPP/H_6_TSPP by taking into account of their vibrational motions of the modes of frequencies, the peaks at 439, 974, 1125, 1193, 1405, 1566 and 1603 cm^−1^ in the IR spectrum of the H_4_TSPP are caused by skeletal deformation of the *meso*-phenyl substitution rings only such as rocking or wagging of its H and vibrational bond stretching, no any contribution comes from the sulfonato groups -SO_3_); (2) the predicted IR peaks are caused by the vibrational motion of the -SO_3_ groups: the strongest IR peak at 980 cm^−1^ in the H_2_TSPP spectrum (which results from the symmetric stretching of O-S-O bonds, ν_s_(O-S-O); a medium intense one at 1151 cm^−1^ (due to asymmetric stretching of O-S-O, ν_a_(O-S-O)); and at a relatively strong peak at 624 cm^−1^ is due to bending deformation of the SO_3_ groups like closing and opening umbrella shape; (3) a relatively strong IR peak at 1160 cm^−1^ in the spectrum of the H_2_TSPP in water used as solvent that is caused by ν_a_(O-S-O) and rocking of CH in phenyl rings, ρ(CH on phenyl); (4) the bond stretching, bending deformation and rocking of CH bonds within sulfonatophenyl substitutes; ν(S-C)), θ(C-C(S)-C) and ρ(CH on phenyl only) produced a very weak IR peak at about 1108 cm^−1^ in spectra of the porphyrins substituted with sulfonatophenyl groups.

The frequency shift predicted is that of 1108 cm^−1^ mode in the sulfonatophenyl-substituted porphyrin which shift to 1049 cm^−1^ in the spectra of phenyl substituted porphyrin; (5) the predicted IR peaks result from due to ν(S-C)/θ(phenyl) and relatively weak w(CH and NH) and out of plane bending (or twisting) deformation of macrocycle (at 748 cm^−1^) and out of plane twisting of the entire molecule, including θ(O-S-O)/w(CH and NH) (at 566 cm^−1^) have very weak intensity, where the θ and w represent the bending deformation and wagging vibration, respectively; (6) the vibrational mode of frequency only result from the H atoms at porphyrin core (NH) caused two IR peaks at low frequency region of 510 and 559 cm^−1^ (due to w(NH)), with very weak intensity; (7) the IR band caused by only rocking of the CβH produced a very weak peak at 1089 cm^‒1^, which is appeared almost at same position for the other systems; (8) the vibrational mode of frequencies caused by the bond stretching (ν), rocking (ρ), wagging (w) and bending deformation (θ) of the C and H atoms within macrocycle are predicted at 751, 825, 848, 1036, 1240, 1299 and 1386 cm^−1^ with a very weak or very weak intensity. These predicted IR features of frequencies are somewhat shifted in spectra of the protonated porphyrins, which are in agreement with experimental observation as provided in Table 4 from refs. [30,31]. It is worth noting that each assignment made in Table 4 was carried out based on the nuclear motion of the atoms within molecule using the Gaussview *visualization* program; (9) the bonding stretching between the *meso*-C atoms (C_m_ within the macrocycle) and C_ϕ_ atom within the phenyl substitution involved in the mode of frequencies (due to ν_s/a_(C_ϕ_-C_m_-C_α_)/θ(C_ϕ_-C_m_-C_α_)/ν_a_(C_β_-C_β_-C_α_)/θ(C_α_-N-C_α_)/ρ(CH) are found at 1220 and 1348 cm^−1^. The peaks caused by symmetric/asymmetric bond stretching, bending deformation or wagging/rocking vibrational motion of the atoms within free base porphyrin (macrocycle) are predicted at 1004, 1460 and 1545 cm^−1^; (10) while in-plane rotational motion of the pyrroline rings, including relatively weak out-of-plane twisting deformation of the phenyl rings produced a weak peak at 439 cm^−1^, a weak peak at 427 cm^−1^ (rocking of phenyl rings (ρ(phenyl) and wagging of macrocycle w(macrocycle and another weak peak at 484 cm^−1^ (caused by twisting of phenyl (phenyl) and w(macrocycle)) are found in the calculated spectrum of H_2_TSSP. Detailed descriptions for each band are given in Table 4.

#### 2.3.1. Isotopic (or Deuteration) Effect on the IR Spectrum

The calculated IR spectra of the molecules studied here clearly indicated that there are not some shifted in the vibrational frequency, but also, the deuterated porphyrin core or sulfonato groups exhibit relatively intense IR peaks in the range of 2630–2660 cm^−1^ (unscaled values) due to N-D bond stretching and around 2711 cm^−1^ (unscaled value) resulting from the O-D bond stretching. The most momentous frequency shifts predicted are those of 540 and 490 cm^−1^ (H_2_TSPP, where all N atoms at porphyrin core are hydrogenated or protonated) shifted to 396 and 366 cm^−1^ in the D_4_HTSPP (indicates that all N atoms at porphyrin core were deuterated) in the range of low frequency region (below 700 cm^−1^) due to wagging of the N-D bond, w(ND).

**Table 4:** Assignments of the IR active modes of frequencies (in cm^−1^) of the meso substituted porphyrin derivatives: TPP (C_2v_ point group), TSPP (C_2v_), H_2_TSPP (C_2v_) and H_6_TSPP (C_2v_) for comparison. The calculations were carried out in water used as solvent at B3LYP/6-311G(d,p) Level of the DFT. Where ∆ν_sc_ stands for scaled vibrational frequencies ((a) ∆ν_sc_ = 0.96∆ν_calc_ +40 as used for the Raman spectra for all molecules studied here) and I_IR_ indicate the calculated IR intensity. In the assignments, the signs ν, θ, ρ and w indicates the bonding stretching, bending deformation, rocking and wagging, respectively. It should be noted that two different scaling factor used for the TPP: (a) ∆ν_sc_ = 0.96∆ν_calc_ +40) and (b) ∆ν_sc_ = 0.976∆ν_calc_. The latter one (b) yields best fitting to observed IR spectrum (from refs. [30,31]) of the TPP only, not for others; however, the scaling factor of ∆ν_sc_ = 0.96∆ν_calc_ +40 yields the best fitting to observed IR spectrum of H_2_TSPP (from ref. [31]) and Raman spectra of the TPP (our previous work, ref. [23]) and H_2_TSPP (present work).

TPP						TSPP			H_2_TSPP				H_6_TSPP			Assignments
Sym	∆ν_sc_.^a^	∆ν_sc_.^b^	I_IR_	∆ν_exp_[30]	∆ν_exp_[31]	Sym	∆ν_sc_.^a^	I_IR_	Sym	∆ν_sc_.^a^	I_IR_	∆ν_exp_[31]	Sym	∆ν_sc_.^a^	I_IR_	
B2	447	414	6	409	406	B2	431	1	B2	439	3	415	B2	439	1	In-plane rotational motion of the pyrroline rings, including relatively weak out-of-plane twisting deformation of the phenyl rings, but no contributions come from the pyrroline rings
						B2	438	13	B2	427	5	445				Rocking of phenyl rings (ρ(phenyl) and wagging of macrocycle w(macrocycle).
B2	447	414	6			B2	475	59	B2	439	3	457	B2	469	1	Out-of-plane bending of phenyl groups only.
B1	553	521	10		516	B1	523	2	A1	484	8		A1	484	8	Twisting of phenyl τ(phenyl) and w(macrocycle)
A1	584	553	2						A1	510	8		A1	487	4	w(NH only)
B2	570	539	2	559	558				B1	559	10		B1	567	10	
						A1	540	8	A1	566	14	560				Out of plane twisting of the molecule and θ(O-S-O)/w(CH and NH)
						B1	541	1	B1	567	6	580				
						B2	627	45	B2	624	63	637	B2	582	39	Due to bending deformation of the SO_3_^−^ groups like closing and opening umbrella shape.
	647	618	2	619	618	A1	657	0.6								In plane bending deformation of phenyl rings, including w(NH and C_β_H only) and out of plane deformation of the macrocycle.
A1	666	636	4	638	636	A1	668	1.0								
B2	688	659	6	658	657											
B2	728	700	43	699	701											w(CH on phenyl) and relatively weak out of plane deformation of the phenyl rings.
						B2	732	6	B2	722	12	715	B2	725	5	w(CH on phenyl) and out of plane deformation of the phenyl rings and macrocycle.
						B2	747	16	B2	748	19	741	B2	739	6	Primarily due to ν(S-C)/θ(phenyl) and relatively weak w(CH an NH) and out of plane bending (or twisting) deformation of macrocycle.
A1	745	716	61			A1	749	10	A1	751	6		A1	753	19	Primarily due to w(C_β_Hs an NH) and out of plane bending (or twisting) deformation of macrocycle, relatively weak out of plane deformation of the phenyl.
B2	757	729	32	727	728											w(C_β_H an NH) and out of plane bending (or twisting) deformation of macrocycle, relatively weak out of plane deformation of the phenyl.
B1	776	749	37			B1	757									w(CH in phenyl and macrocycle) and out of plane bending (or twisting) deformation of phenyl rings the macrocycle.
B2	776	749	21	746	748	B2	767	1								
													A1	769	32	Mainly due to ν(S-O(H)), including w(C_β_Hs an NH) and out of plane bending (or twisting) deformation of macrocycle, relatively weak out of plane deformation of the phenyl.
													B2	773	66	
A1	815	788	3	785	788	B1	823	7	B1	825	2	800	B1	826	3	W(C_β_Hs and NH) and out of plane bending deformation of macrocycle
A1	829	802	100	798	799	A1	829	20	A1	848	23	854	A1	852	15	
B1	894	869	10	875	871	B1	896	1								θ(N-C_α_-C_β_ and N-C_α’_-C_β’_ in the same phase)/θ(C_m_-C_α_-N)/θ(C_α_-C_m_-C_α_)/θ(phenyl)/ρ(C_β_H)
B1	985	960	91	964	962	B1	968	5	B1	974	1	966	B1	998	2	W(CH on phenyl)
						B2	980	63	B2	980	100	984				νs(O-S-O)
B2	1001	977	34			B2	1002	5	B2	1004	2	1012	B2	1004	9	θ(N-C_α_-C_β_ and N-C_α’_-C_β’_ in the same phase)/θ(C_m_-C_α_-N)/θ(C_α_-C_m_-C_α_)/θ(phenyl)/ρ(C_β_H)
A1	1020	996	0.	979	980	A1	1020	0.	A1	1036	2	1039	A1	1035	1	Expansion of the pyrrole/pyrroline groups along N(H)…N(H) direction due to ν(C_α_-C_β_), leading to macrocycle getting rectangular shape instead of square shape.
B1	1014	991	11													θ(C-C-C in phenyl)
B2	1016	993	33	999	1002	B2	1016	1								ρ(C_β_H)/θ(C-C-C in phenyl)/θ(C_m_-C_α_-N)
B1	1049	1026	9	1031	1032				B1	1033	<1					ρ(CH in phenyl)/θ(C-C-C in phenyl)
B1	1087	1065	9	1069	1072				B1	1089	1					ρ(C_β_H)
A1	1094	1072	10													ρ(C_β_H and CH on phenyl).
						B1	1109	21	B1	1108	11		B1	1107	3	ν(C-S)/θ(C-C(S)-C)/ρ(CH on phenyl only)
						A1	1116	14	A1	1125	6	1125	A1	1133	1	ρ(CH on phenyl only)
						B2	1146	64	B1	1151	30					νa(O-S-O)
						A1/B1	1156	100	A1/B1	1160	35	1188a	A1/B1	1151	100	νa(O-S-O)/ρ(CH on phenyl)
B2	1180	1159	38		1155	B2	1180	8								νa(C-N-C)/θ(C-N-C)/ ρ(C_β_H)
B2	1189	1168	4	1174	1176	B2	1188	0.	B2	1193	9		B2	1200	3	ρ(CH on phenyl only)
B1	1204	1183	25	1187	1187	B1	1204	4								ρ(NH) and relatively weak θ(whole molecule)
B2	1224	1203	13	1211	1211	B2	1224	2	B2	1220	14	1218	B2	1221	2	νs(C-N-C)/νs(C_ϕ_-C_m_-C_α_)/ρ(NH and CH)
B1	1235	1215	17	1220	1219	B1	1235	3	B1	1240	12		B1	1241	7	νa(C-N-C)/ρ(NH and C_β_H)
B1	1262	1242	9	1247	1251	B1	1262	2	B1	1299	11		B1	1301	2	ν(C_β_-C_α_)/θ(C-N-C)/ρ(C_β_H and NH).
B1	1355	1337	47	1348	1351	B1	1354	8	B1	1348	1	1350	B1	1387	1	νa(C_β_-C_β_-C_α_)/ θ(C_α_-N-C_α_)/νa(C_ϕ_-C_m_-C_α_)/θ(C_ϕ_-C_m_-C_α_)/ρ(CH).
B1	1373	1356	24	1359	1358	B1	1373	4	B1	1386	5	1384	B1	1387	1	νa(C-N-C)/ρ(C_β_H and NH).
B2	1411	1394	22	1400	1400	B2	1411	4								ν(C_β_-C_α_)/ν(C_α_-C_m_) which also leading to νa(C_β_-C_α_-C_m_), including ρ(C_β_H).
A1	1451	1435	12	1459	1437	B2	1399	2	B1	1405	9	1395	B1	1407	6	ρ(CH on phenyl), including relatively weak νs(C-C-C_ϕ_)
B2	1482	1466	82	1471	1471	B2	1482	16	B2	1460	62	1472	B2	1467	17	Ν(C_β_-C_β_)/ν(C_α_-C_m_) that leading to θ(C-N-C)
B1	1492	1486	7	1488	1493											ν(C_α_-C_m_)/ν(C_β_-C_β’_)/νa(C_α_-NH-C_β_), including ρ(CH) on the macrocycle only.
B1	1568	1554	67		1555	B1	1567	10	B1	1545	1	1554	B1	1545	3	
B1	1588	1574	1	1573	1575				B2	1566	1	15581564				νa( C-C-C) within phenyl rings and ρ(H on phenyl)
B2	1612	1598	30	1595	1595	B2	1607	2	B1	1603	8	15921597	B2	1604	1	ν(C-C)/ρ(CH) within phenyl rings, including θ(C-C-C in phenyl).

In the high or mid frequency region, when the deuterium atom(s) is involved in the vibrational mode of frequency that lead to a red shift in the frequency as much as up to 10 cm^−1^ for the D_2_TSPP spectrum. The deuteration also have an effect on the relative intensity of the IR peaks in some cases, see Figure 4. These shift in the high frequency shift for the D_2_TPyr, D_4_TPyr and D_4_TPP are more significant than these for D_2_TSPP and D_4_TSPP. This observation indicates that above the low frequency region, the frequency shift due to deuteration decreases with increasing size of the substituent group.

#### 2.3.2. Solvent Effect on the IR Spectrum

Also, the solvent effect on the IR spectrum of the H_2_TSPP was investigated. We used dimethylsulfoxide (DMSO) and toluene instead of water as solvent beside vacuum (gas-phase) in the calculations. The calculations indicated that, below 1100 cm^−1^, there is no any significant frequency shift in the peak positions, but there are above 1100 cm^−1^. For instance, while the IR peak centered at around 1200 cm^−1^ in gas phase is shifted to 1188 cm^−1^ (toluene), 1166 cm^−1^ (DMSO), and 1160 cm^−1^ (water), the IR peaks centered around 1477 and 1453 cm^−1^ in gas phase spectrum that are shifted to 1490 and 1468 cm^−1^ (toluene), 1497 and 1480 cm^−1^ (DMSO), and 1499 and 1481 cm^−1^ (water). These results indicate that the IR features, in high energy region, of the porphyrin and its derivatives, at least for H_2_TSPP, are sensitive to its environment. For that reason, the experimentally measured IR spectrum may exhibits frequency shifts in the spectral location of peaks in the different environments; accordingly we need to be careful about the assignment of the IR features.

### 2.4. Calculated Electronic Spectra of Porphyrin and Its Derivatives

**Figure 5:**
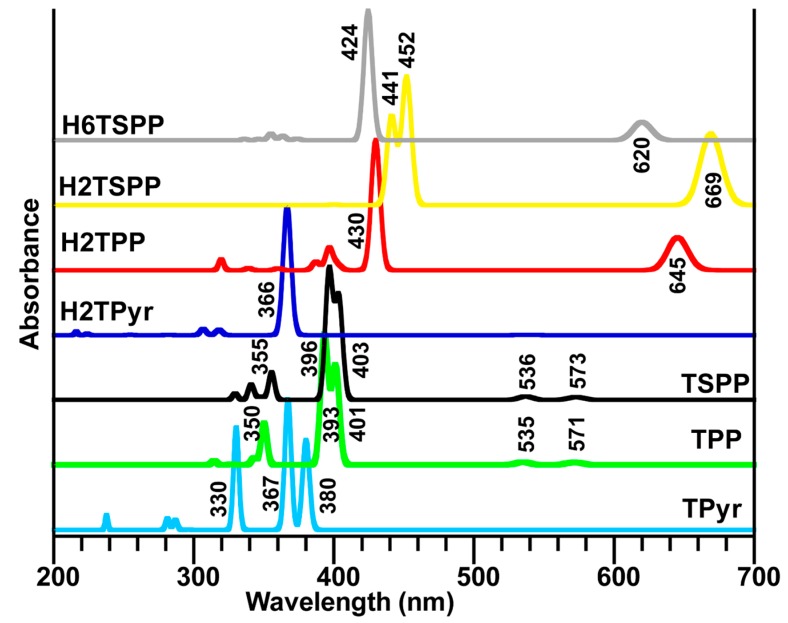
Calculated dipole allowed electronic transitions of porphyrin derivatives in water used as a solvent in the calculations at the TD-B3LYP/6-31G(d,p) level of the TD-DFT: free-base porphyrin (TPyr), *meso*-tetraphenylporphyrin (TPP), dianionic meso-tetrakis(*p*-sulfonatophenyl)porphyrin (TSPP), and protonated-TPyr (H_2_TPyr), protonated-TPP (H_2_TPP), protonated TSPP (H_2_TSPP) and dicationic TSPP (H_6_TSPP).

Excited states are not only important to processes such as for electronics, photosynthesis, but they also play a crucial role in the fields of renewable energy, material design and medicine. Therefore, we investigated vertical electronic transitions of the porphyrin derivatives studied here for both singlet triplet states (up to 24 singlet and 24 triplet states) that are provided in Figure 5 and Table 5. 

The calculations mainly produced a strong electronic absorption band in the range of 450–360 nm and a few weak or very weak electronic transition below and above the strong band. The strongest band is known as Soret band (B-band, in the range of about 400–450 nm) and weaker bands (in longer wavelength region, about 450–750 nm) are known as Q-bands. The electronic spectra of porphyrin and its derivatives studied here discussed below. It is worthy to note that the percentage given in parenthesis indicates the contributions from the different HOMO (H)→LUMO (L) transitions to a desired electronic transitions. The minor contributions are not shown here.

**Table 5:** The selected values of the calculated singlet-singlet (**S_0_→S_n_**) and singlet-triplet (**S_0_→T_n_**) vertical electronic transitions for the TPyr, H_2_TPyr, TPP, H_2_TPP, TSPP, H_2_TSPP, and H_6_TSPP (in water used as a solvent) at TD-B3LYP/6-31G(d,p) level of the TD-DFT. The percentages in the parenthesis indicate the contributions from the different HOMO(H)→LUMO(L) transitions to a desired electronic transitions and the minor contributions are not provided here.

**TPyr/S_0_→S_n_**						**S_0_→T_n_**				
**S_n_**	**(eV)**	**(nm)**	**F**	**Sym**	**Major Contribs**	**T_n_**	**(eV)**	**(nm)**	**Sym**	**Major Contribs**
1	2.30	540	0.0005	B1U	H−1− > L+1 (40%),	1	1.51	822	B2U	H−1− > L (21%),
					H− > L (59%)					H− > L+1 (79%)
2	2.45	506	0.0003	B2U	H−1− > L (47%),	2	1.82	682	B1U	H− > L (94%)
					H− > L+1 (53%)					
3	3.26	380	0.8144	B1U	H−3− > L (22%),	3	2.04	608	B2U	H−1− > L (78%),
					H−1− > L+1 (48%),					
					H− > L (29%)					H− > L+1 (22%)
4	3.38	367	1.1911	B2U	H−1− > L (50%),	4	2.07	598	B1U	H−1− > L+1 (94%)
					H− > L+1 (47%)					
5	3.44	360		B3G	H−2− > L (98%)	7	2.90	428	B3G	H−2− > L (88%)
6	3.66	339		AG	H−2− > L+1 (99%)	8	2.96	419	B1U	H−3− > L (86%)
7	3.76	330	0.6934	B1U	H−3− > L (76%),	9	3.15	393	AG	H−2− > L+1 (93%)
					H−1− > L+1 (12%),					
					H− > L (12%)					
8	3.76	330	0.2479	B2U	H−3− > L+1 (93%)	11	3.33	373	B3G	H−8− > L+1 (16%),
										H− > L+2 (72%)
16	4.33	287	0.0914	B2U	H−5− > L+1 (97%)	13	3.39	366	B2U	H−3− > L+1 (96%)
18	4.41	281	0.1037	B1U	H−5− > L (99%)	15	3.61	343	AG	H−8− > L (26%),
										H−1− > L+2 (68%)
23	5.22	237	0.1338	B1U	H−2− > L+2 (98%)	16	3.64	340	B3G	H−4− > L+1 (79%)
**H_2_TPyr/S_0_→S_n_**						**S_0_→T_n_**				
**S_n_**	**(eV)**	**(nm)**	**F**	**Sym**	**Major contribs**	**T_n_**	**(eV)**	**(nm)**	**Sym**	**Major contribs**
1	2.31	538	0.0007	E	H−1− > L+1 (48%),	1	1.63	763	E	H−1− > L+1 (30%),
					H− > L (52%)					H− > L (70%)
2	2.31	538	0.0007	E	H−1− > L (48%),	2	1.63	763	E	H−1− > L (30%),
					H− > L+1 (52%)					H− > L+1 (70%)
3	3.39	366	1.4554	E	H−1− > L+1 (52%),	3	1.96	632	E	H−1− > L+1 (69%),
					H− > L (48%)					H− > L (31%)
4	3.39	366	1.4554	E	H−1− > L (52%),	4	1.96	632	E	H−1− > L (69%),
					H− > L+1 (48%)					H− > L+1 (31%)
7	3.90	318	0.0597	E	H−5− > L+1 (45%),	7	3.23	384	B1	H−3− > L+1 (28%),
										H−2− > L (28%),
					H−4− > L+1 (53%)					H− > L+2 (31%)
8	3.90	318	0.0597	E	H−5− > L (45%),	8	3.32	374	E	H−3− > L+1 (44%),
					H−4− > L (53%)					H−2− > L (44%)
11	4.05	306	0.0695	E	H−5− > L+1 (54%),	9	3.37	367	E	H−5− > L+1 (42%),
					H−4− > L+1 (45%)					H−4− > L+1 (48%)
12	4.05	306	0.0695	E	H−5− > L (54%),	10	3.37	367	E	H−5− > L (42%),
					H−4− > L (45%)					H−4− > L (48%)
**TPP/S_0_→S_n_**						**S_0_→T_n_**				
**S_n_**	**(eV)**	**(nm)**	**F**	**Sym**	**Major contribs**	**T_n_**	**(eV)**	**(nm)**	**Sym**	**Major contribs**
1	2.17	571	0.0337	B2	H−1− > L+1 (32%),	1	1.40	884	B1	H−1− > L (16%),
					H− > L (67%)					H− > L+1 (84%)
2	2.32	535	0.0359	B1	H−1− > L (37%),	2	1.66	745	B2	H− > L (98%)
					H− > L+1 (63%)					
3	3.09	401	1.2834	B2	H−3− > L (10%),	3	1.99	623	B1	H−1− > L (84%),
					H−1− > L+1 (62%),					
					H− > L (27%)					H− > L+1 (15%)
4	3.16	393	1.6972	B1	H−1− > L (62%),	4	2.06	602	B2	H−1− > L+1 (97%)
					H− > L+1 (37%)					
6	3.54	350	0.5462	B2	H−3− > L (87%)	5	2.84	436	A2	H−2− > L (88%)
8	3.62	343	0.0909	B1	H−3− > L+1 (98%)	6	2.90	428	B2	H−3− > L (82%)
19	3.95	314	0.0267	B1	H−10− > L (39%),	7	3.08	403	A1	H−2− > L+1 (91%)
					H−8− > L+1 (57%)					
20	3.95	314	0.0216	B2	H−14− > L (15%),	8	3.15	393	A2	H−16− > L+1 (10%), H− > L+2 (74%)
					H−11− > L (56%),					
					H−10− > L+1 (14%),					
					H−8− > L (10%)					
**H_2_TPP/S_0_→S_n_**						**S_0_→T_n_**				
**S_n_**	**(eV)**	**(nm)**	**F**	**Sym**	**Major contribs**	**T_n_**	**(eV)**	**(nm)**	**Sym**	**Major contribs**
1	1.92	645	0.304	A'	H−1− > L+1 (16%),	1	1.22	1020	A"	H− > L+1 (98%)
					H− > L (84%)					
2	1.92	645	0.3039	A"	H−1− > L (16%),	2	1.22	1020	A'	H− > L (98%)
					H− > L+1 (84%)					
3	2.89	430	1.2029	A'	H−1− > L+1 (74%),	3	2.02	615	A"	H−1− > L (95%)
					H− > L (14%)					
4	2.89	430	1.2026	A"	H−1− > L (74%),	4	2.02	615	A'	H−1− > L+1 (95%)
					H− > L+1 (14%)					
10	3.13	396	0.2053	A"	H−5− > L (89%)	5	2.67	464	A"	H−3− > L (13%),
										H−2− > L+1 (13%),
										H− > L+2 (56%)
11	3.13	396	0.2056	A'	H−5− > L+1 (89%)	6	2.76	449	A'	H−7− > L+1 (16%),
										H−6− > L (16%),
										H−3− > L+1 (29%),
										H−2− > L (29%)
15	3.20	387	0.078	A"	H−8− > L (80%)	7	2.88	431	A'	H−3− > L+1 (46%),
										H−2− > L (46%)
16	3.20	387	0.0778	A'	H−8− > L+1 (80%)	8	2.88	430	A"	H−3− > L (46%),
										H−2− > L+1 (46%)
21	3.45	360	0.0351	A'	H−10− > L (12%),	9	2.93	424	A'	H−5− > L+1 (82%)
					H−9− > L (80%)					
22	3.66	339	0.039	A"	H−10− > L+1 (13%),	10	2.93	424	A"	H−5− > L (82%)
					H−9− > L+1 (80%)					
**TSPP/S_0_→S_n_**						**S_0_→T_n_**				
**S_n_**	**(eV)**	**(nm)**	**F**	**Sym**	**Major contribs**	**T_n_**	**(eV)**	**(nm)**	**Sym**	**Major contribs**
1	3.88	319	0.1998	A'	H−10− > L (78%), H−9− > L (11%)	11	2.94	421	A"	H−7− > L (17%),
										H−6− > L+1 (17%),
										H−3− > L (13%),
										H−2− > L+1 (13%),
										H− > L+2 (31%)
5	2.16	573	0.0419	B2	H−1− > L+1 (32%),	1	1.40	884	B1	H−1− > L (16%),
					H− > L (67%)					H− > L+1 (84%)
6	2.31	536	0.0506	B1	H−1− > L (36%),	2	1.67	744	B2	H− > L (97%)
					H− > L+1 (64%)					
10	3.07	403	1.4382	B2	H−1− > L+1 (62%),	3	1.99	624	B1	H−1− > L (84%),
					H− > L (28%)					H− > L+1 (15%)
11	3.13	396	1.8378	B1	H−1− > L (62%),	4	2.05	604	B2	H−1− > L+1 (97%)
					H− > L+1 (36%)					
36	3.49	355	0.3924	B2	H−10− > L (83%)	5	2.84	437	A2	H−9− > L (49%),
										H−7− > L (39%)
38	3.56	348	0.0392	B1	H−10− > L+1 (28%),	6	2.89	429	B2	H−11− > L (36%),
					H−8− > L (69%)					H−10− > L (48%)
47	3.64	341	0.2178	B2	H−11− > L (83%)	7	3.07	404	A1	H−9− > L+1 (39%),
										H−7− > L+1 (51%)
48	3.77	329	0.0936	B1	H−11− > L+1 (82%),	8	3.14	395	A2	H− > L+2 (70%)
					H−10− > L+1 (11%)					
**H_2_TSPP/S_0_→S_n_**						**S_0_→T_n_**				
**S_n_**	**(eV)**	**(nm)**	**F**	**Sym**	**Major contribs**	**T_n_**	**(eV)**	**(nm)**	**Sym**	**Major contribs**
1	1.85	669	**0.4223**	B2	H−5− > L+1 (13%),	1	1.17	1056	B2	H− > L (97%)
					H− > L (87%)					
2	1.85	669	**0.4138**	B1	H−5− > L (13%),	3	2.34	530	A1	H−3− > L+1 (47%),
					H− > L+1 (87%)					H−2− > L (52%)
4	2.35	528	**0.0001**	A1	H−3− > L+1 (47%),	5	2.34	529	B1	H−4− > L+1 (47%),
					H−2− > L (53%)					H−1− > L (52%)
5	2.35	528	**0.0005**	B2	H−4− > L (53%),	7	2.01	618	B1	H−5− > L (95%)
					H−1− > L+1 (47%)					
10	2.40	518	**0.0001**	B2	H−4− > L (47%),	9	2.39	518	A1	H−3− > L+1 (53%),
					H−1− > L+1 (53%)					H−2− > L (47%)
12	2.74	452	**0.7601**	B1	H−6− > L (79%),	13	2.51	494	A2	H−8− > L+1 (34%),
										H−7− > L (35%),
					H−5− > L (15%)					H− > L+2 (23%)
13	2.75	451	**0.7369**	B2	H−6− > L+1 (83%),	14	2.62	474	B1	H−6− > L (90%)
					H−5− > L+1 (12%)					
16	2.81	441	**0.5315**	B2	H−9− > L (17%),	17	2.65	467	A1	H−8− > L (46%), H−7− > L+1 (46%)
					H−6− > L+1 (16%),					
					H−5− > L+1 (57%)					
18	2.81	441	**0.5073**	B1	H−9− > L+1 (16%),	18	2.69	462	A2	H−8− > L+1 (47%), H−7− > L (47%)
					H−6− > L (19%),					
					H−5− > L (54%)					
19	2.97	417	**0.0013**	B1	H−13− > L (14%),	19	2.72	455	A2	H−8− > L+1 (13%),
										H−7− > L (12%),
					H−10− > L+1 (74%)					H− > L+2 (63%)
22	3.10	400	**0.0081**	B2	H−13− > L+1 (12%),	20	2.96	419	A2	H−12− > L+1 (43%),
					H−10− > L (82%)					H−11− > L (47%)
**H_6_TSPP/S_0_→S_n_**						**S_0_→T_n_**				
**S_n_**	**(eV)**	**(nm)**	**F**	**Sym**	**Major contribs**	**T_n_**	**(eV)**	**(nm)**	**Sym**	**Major contribs**
1	2.00	620	0.2467	B2	H−1− > L+1 (23%),	1	1.31	946	B2	H− > L (96%)
					H− > L (77%)					
2	2.00	619	0.2377	B1	H−1− > L (23%),	2	1.31	945	B1	H− > L+1 (96%)
					H− > L+1 (77%)					
3	2.92	424	1.7153	B1	H−1− > L (74%),	3	1.94	639	B1	H−1− > L (94%)
					H− > L+1 (23%)					
4	2.92	424	1.7145	B2	H−1− > L+1 (75%),	4	1.94	638	B2	H−1− > L+1 (94%)
					H− > L (22%)					
7	3.32	374	0.0199	B2	H−4− > L (92%)	5	2.74	452	A2	H− > L+2 (72%)
8	3.32	373	0.0240	B1	H−4− > L+1 (92%)	6	3.00	413	A1	H−7− > L (26%),
										H−6− > L+1 (26%),
										H−3− > L (13%),
										H−2− > L+1 (18%)
11	3.41	363	0.0560	B1	H−5− > L (88%)	7	3.08	403	A2	H−3− > L+1 (37%),
										H−2− > L (40%)
12	3.42	363	0.0614	B2	H−5− > L+1 (88%)	8	3.09	401	A1	H−3− > L (36%),
										H−2− > L+1 (34%),
										H−1− > L+2 (14%)
15	3.50	355	0.0822	B1	H−8− > L (94%)	9	3.16	393	B2	H−5− > L+1 (16%),
										H−4− > L (66%)
16	3.50	355	0.0918	B2	H−8− > L+1 (94%)	10	3.16	393	B1	H−5− > L (17%),
										H−4− > L+1 (65%)
20	3.59	346	0.0365	B2	H−10− > L (10%), H−9− > L (84%)	11	3.20	387	A2	H−12− > L+1 (11%),
										H−11− > L (12%),
										H−7− > L+1 (29%),
										H−6− > L (30%)
21	3.69	336	0.0363	B1	H−10− > L+1 12%),	12	3.23	384	B1	H−8− > L (66%)
					H−9− > L+1 (81%)					

#### 2.4.1. The Electronic Spectra of the TPyr and Protonated-TPyr (H_2_TPyr)

**Figure 6:**
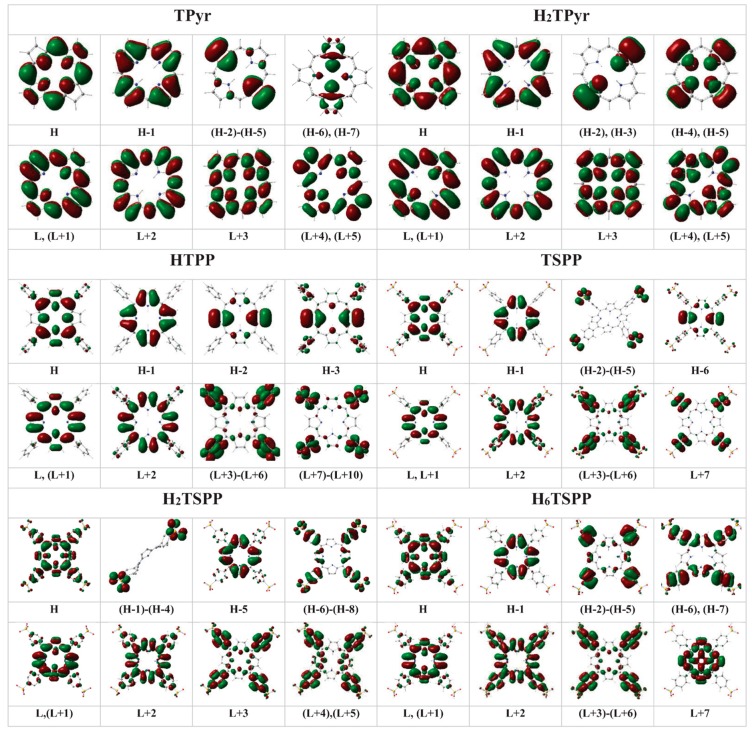
The plotted electron densities in the HOMOs (H) and LUMO (Ls) of: free-base porphyrin (TPyr), *meso*-tetraphenylporphyrin (TPP), dianionic meso-tetrakis(*p*-sulfonatophenyl)porphyrin (TSPP), and protonated-TPyr (H_2_TPyr), protonated-TPP (H_2_TPP), protonated TSPP (H_2_TSPP) and dicationic TSPP (H_6_TSPP) molecules.

The electronic spectrum of the TPyr molecule exhibited two weaker electronic transitions below the Soret band (S): S_0_→S_1_ (B_1u_), H−1→L+1 (40%) and H→L (59%), at 540 nm (with oscillator strength, f = 0.0005) and S_0_→S_2_ (B_2u_), H−1→L+1 (47%) and H→L+1 (53%), at 506 nm (f = 0.0003); two strong electronic transitions in the range of Soret band (B-band): S_0_→S_4_ (B_2u_), results from H−1→L (50%), and H→L+1 (47%), at 367 nm (f = 1.1911), and S_0_→S_3_ (B_1u_), originates from H−3→L (23%), H−1→L+1 (49%) and H→L (30%), at 380 nm (f = 0.8144) beside a few weaker bands: S_0_→S_7_ (B_1u_), H−3→L (76%), H−1→L+1 (12%) and H→L (12%), at 330 nm (f = 0.6934); S_0_→S_8_ (B_2u_), H−3→L+1 (93%), at 330 nm (f = 0.2479); S_0_→S_13_ (B_3u_), H−7→L+1 (99%), at 296 nm (f = 0.0019); S_0_→S_16_ (B_2u_), H−5→L+1 (97%), at 287 nm (f = 0.0914); S_0_→S_18_ (B_1u_), H−5→L (99%), at 281 nm (f = 0.1037); and S_0_→S_23_ (B_1u_), H−2→L+2 (98%), at 237 nm (f = 0.1338). Where H−n and L+n molecular orbitals are not only pure π/σ and π*/σ* molecular orbitals (MOs) as seen in Figure 6, they also include nonbinding atomic orbitals (AOs) in particular cases such as: H is a symbol of the π(C_β_-C_β_/C_m_-C_α_)+n(N); similarly, H−1: π(C_β_-C_α_), H−2: π(C_α_-N-C_α_/C_β_-C_β_); H−3: π(C_α_-N-C_α_/C_β_-C_β_)+n(minor, N/C_m_); H−4/H+5: π(C_β_-C_β_)+n(N); H−6/H−7: n(N)+σ(minor; C_β_-C_α_); L/L+1: π*(C_β_-C_β_/C_β_-C_α_/C_m_-C_α_)+n(minor; N); L+2: π*(C_β_-C_α_)+n(C_m_); L+3: π*(C_β_-C_β_/C_m_-C_α_)+n(N/C_α_); L+4/L+5: π*(C_β_-C_β_/C_m_-C_α_)+n(N/C_α_/C_β_); L+6/L+7: σ*(H-H/macrocycle)+n(N); and L+8: n*(C). 

The electronic spectrum of the protonated TPyr (H_2_TPyr) exhibited Q- and B-bands originate from the S_0_→S_1/2_ (E), H−1→L (48%) and H→L+1(52%), at 538 nm (f = 0.0007); S_0_→S_3/4_ (E), H−1/→L+1 (53%) and H→L (49%), at 366 nm (f = 1.4554); S_0_→S_22/23_ (E), H−5→L+1 (45%) and H−4→L+1 (53%), at 318 nm (f = 0.0597); S_0_→S_11/12_ (E), H−5→L+1 (54%) and H−4→L+1 (45%), at 306 nm (f = 0.0695); S_0_→S_15_ (B_2_), H−1→L+2 (78%), at 282 nm (f = 0.0049); S_0_→S_18_ (B_2_), H−7→L (10%), H−6→L+1 (10%), H−1→L+2 (11%) and H→L+3 (66%), at 224 nm (f = 0.0337); S_0_→S_22/23_ (E), H−3→L+2 (95%), at 216 nm (f = 0.0407). Where the H: π(C_β_-C_β_ and C_α_-C_m_-C_α_)+n(N); H−1: π(C_α_-C_β_); H−2/H−3: π(C_α_-C_β_)+n(N); H−4/H−5: π(C_β_-C_β_)+n(N); H−6/H−7: π(C_β_-C_α_-C_m_)+n(minor, N); H−8: π(C_α_-C_m_-C_α_); L/L+1: π(C_β_-C_α_)+n(minor, N); L+2: π(C_β_-C_α_) + n(C_m_); L+3: π(C_β_-C_β_)+n(N/C_α_/C_m_); L+4: π(C_β_-C_β_ and C_α_-C_m_)+n(N/C_α_/C_m_). Comparing the electronic spectrum of TPyr with that of H_2_TPyr, while the TPyr exhibit three strong bands at 380, 360 and 330 nm (see Figure 5 and Table 5), H_2_TPyr showed a strong band at 366 nm only in the Soret band region. In the Q-band region, the electronic band positions are shifted from 540 and 506 nm in TPyr to 539 nm (doubly degenerated) in H_2_TPyr.

Furthermore, the calculations produced twenty-four triplet states in the range of 822–249 nm for the TPyr molecule (in water used as solvent). There are two triplet states at 373 nm with symmetry B_3g_, T_8_(B_3g_), and at 366 nm (T_9_(B_2u_)) that are almost overlap with the strongly dipole allowed singlet electronic state at 367 nm (S_4_(B_2u_)). This finding indicate that there is no only possibility of the internal-conversion (IC) process between the S_4_(B_2u_ at 376 nm) and S_1_(B_1u_ at 540 nm) and S_2_(B_2u_ at 506 nm), but also, the possibility of the inter-system-crossing (ISC) process through potential energy surface (PES) touching or strong vibrational coupling between S_4_ and T_8/9_ electronic states in the excited state.

For the protonated TPyr (H_2_TPyr) molecules exhibited similar futures such as: the IC between the S_3/4_ (the strongest bands or B-band) at 366 nm (with the symmetry E) and the S_1_ (at 538 nm with symmetry E); the ISC process between the S_1_ (at 538 nm with symmetry E) and T_7/8_ (triplet states at 367 nm with the symmetry E), see Table 5.

#### 2.4.2. The Electronic Spectra of TPP and H_2_TPP

While the TPP molecule exhibited two weak peaks at 571 nm (S_0_→S_1_, f = 0.00337) and 535 nm (S_0_→S_2_, f = 0.0359) in Q-band region, the H_2_TPP produced only a double degenerated peak that is red shifted to 645 nm (S_0_→S_1/2_, f = 0.3040). In the B-band region, two strong peaks at 401 and 393 nm (S_0_→S_3/4_, f = 1.2834/1.6972) in the TPP absorption spectrum and a doubly degenerated band at 430 nm (S_0_→S_3/4_, f = 1.209) in the H_2_TPP. Both spectra exhibited a few weak and very weak allowed electronic transitions in the high energy region as seen in Table 5 and Figure 5. Jiang *et al*. [32] measured absorption and EPR spectra of some porphyrins (TPP and derivatives) and metalloporphyrins compounds. The measured absorption spectrum of the TPP produced an intense electronic transition at 417 nm (B-band) and several Q-bands with weak intensity at 514, 550, 593 and 646 nm. As seen in Table 5 and Figure 5, while these observed electronic bands are consistent with the calculated values as mentioned above, the electronic transition at 646 nm is not. The calculations did not even produce any dipole forbidden singlet-singlet electronic transition with wavelength longer than 571 nm, but, a weak singlet-singlet transition was predicated at 645 nm for the protonated TPP (H_2_TPP) molecule. This observation suggest that the H_2_TPP might be formed in the TPP solution with a low concentration and high pH value (pH > 7) or there might be singlet-triplet transition (predicted at 623 nm) through the intensity borrowing process. The authors have also reported that both steric hindrance and electron effects of the functional groups influenced the UV-vis absorption indicate that the TPP of Soret bands of the studied *para*-substituted *meso*-tetraphenylporphine derivatives moved slightly toward short wavelength (3–5 nm). This experimental observation is consistent with our calculations, for instance, when molecular system going from the TPyr, TPP to TSPP or from the H_2_TPyr, H_2_TPP to H_2_TSPP (see Figure 5 or Table 5).

Additionally, the results of the calculations of the TPP (in solution/water) indicated the possibility of the IC process between the B-bands (S_3_ at 401 nm (with symmetry B_2_), S_4_ at 393 nm (B_1_) and S_6_ at 350 nm (B_2_)) and S_1_ (at 571 nm (B_2_)/S_2_ (at 535 nm (B_1_); the ISC process between the B-bands (S_3_ at 401 nm (B_2_) and T_7_ (at 403 nm (A_1_))/T_8_ (at 393 nm (A2)/T_11_ (at 348 nm (A_2_) through surface touching since the these singlet and triplet states are almost overlapping, based on the calculations.

For the protonated TPP molecule (H_2_TPP), the IC process between the B-bands (S_3/4_ at 430 nm (A' and A")) and S_1/2_ (at 645 nm (A' and A")), the ISC process between the doubly degenerated Q-band (S_1/2_ at 645 nm (A' and A")) and T_6_ (at 449 nm with the symmetry A') and between the degenerated B-band (S_3/4_ at 430 nm (A' and A")) and T_7/8_ (at 431 and 430 nm (A' and A")).

#### 2.4.3. Calculated Electronic Spectra of the TSPP, H_2_TSPP and H_6_TSPP

The experimentally observed absorption spectra of the free base TSPP exhibited an intense band (S-band, also known as B-band) at ~410 nm and several weak bands (known as Q-bands) at about 515, 550, 580, 640 nm by Akins *et al.* [21] and Zhang *et al.* [31]. Also, the authors have reported that the absorption spectrum of the dianionic TSPP (here we referred as H_2_TSPP) displayed the S-band at 432 nm and Q-bands at 540, 580 and 642 nm. Also, Akins and coworkers have measured fluorescence spectra of free-base TSPP (pH = 12) and monomeric H_2_TSPP (pH = 4.5), and aggregate TSPP. Their measured fluorescence spectra at excitation 412, 432, and 488 nm (the B-bands for the TSPP, H2TSPP and the aggregated-TSPP, respectively) showed that emission arises from the lowest excited electronic singlet state (S_1_) associated with the Q-bands, while excitation might be to the S_2_ state, the higher excited state associated with the B-band. (The fluorescence spectrum at excitation 412 nm exhibited a broadened and red degraded peak at 642 nm and a weak one at 702 nm; at the excitation 432 nm, they observed an intense broadened peak with a shoulder at 655 nm. These experimental clearly indicate an internal conversion (CI) from the B-band to the Q-band [21].

In the Q-band region, while the calculated exhibited two weak transitions at 573 nm (S_0_→S_1_ with symmetry B_2_ and f = 0.0419) and at 536 nm (S_0_→S_2_ with symmetry B_1_, f = 0.0506) in the TSPP spectrum; and at 669 nm (S_0_→S_1/2_ with symmetries B_2_ and B_1_, f = 0.4223/0.4138) and 528 nm (S_0_→S_4/5_ with symmetries A_1_ and B_2_, f = 0.0001/0.0005), and at 518 nm (S_0_→S_10_, B_2_ symmetry and f = 0.0001) in the H_2_TSPP; however, the H_6_TSPP (or dicationic TSPP) exhibited only one doubly degenerated weak electronic band at 620/619 nm (S_0_→S_1/2_ with symmetries B_2_ and B_1_, f = 0.2467/0.2377). In the B-band (Soret band) region, the calculations revealed a strong band at 403 (S_0_→S_3_ with B_2_ symmetry and f = 1.4382), and at 396 nm (S_0_→S_4_ with symmetry B_1_ and f = 1.8378) for the TSPP; a strong band at 452/451 nm (S_0_→S_12/13_, B_1 _and B_2_ symmetries, f = 0.7601/0.7369) and a medium intense band at 441 nm (S_0_→S_16_, B_2_ symmetry and f = 0.5315) for the H_2_TSPP; and a strong transition at 424 nm (S_0_→S_3/4_, B_1_ and B_2_ symmetries and f = 1.7153/1.7145) for H_6_TSPP. Also, many weak electronic transitions are found above these strong bands (see Table 5). The predicted B-bands and Q-bands for these molecules are compatible with the experimental data [21,31].

The predicted possible IC and ISC processes: for the TSPP, the IC (internal conversion) can happen from S_3_ (at 403 nm) to S_1_ (at 573 nm) and S_2_ (at 536 nm). The ISC (intersystem crossing) process may take place from the S_3_ (at 403 nm)/S_4_ (at 396 nm) to the T_7_ (at 404 nm)/T_8_ (at 395 nm), but not, take place between the Q-bands and any triplet states, based on the calculations. For the H_2_TSPP (or dianionic TSPP): the IC process may take place from the B-bands, S_12/13_(at 452/451 nm)/S_16_ (at 441 nm), to Q-bands S_10_ (at 518 nm)/S_4/5_(at 528 nm)/S_1/2_(at 669 nm), which is consistent with experimental observation (at 665 nm) [21]; the ISC process may occur from the Q-bands to the triplet states such as S_4/5_(at 528 nm) to T_3,4,5,6_(at 530 and 529 nm), and S_10_ (at 518 nm) to T_15,16,17,18_(at 518 nm). In the high energy region, the ISC may originate from B-bands to triplet states: S_12/13_(at 452/451 nm) to the T_19_ (at 455 nm). Likewise, for the H_6_TSPP dicationic molecule: the IC process may happen between the B-bands (S_3/4_(at 424 nm)) and Q-bands (S_1/2_(at 620/619 nm)); the ISC process between the Q-bands and triplet states such as from S_1/2_(at 620/619 nm) to T_3/4_(at 639 and 638 nm). In order to the ISC process happens between the B-band and the closest triplet state(s) (T) that is expected very strong vibrational coupling in the excited state since the energy separation between the S_3/4_(B_1_/B_2_) at 424 nm and the closest triplet states at 452 nm (T_5_(A_2_) and at 413 nm (T_6_(A_1_) are 28 nm (−0.18 eV or −1652 cm^−1^) and −13 nm (0.08 eV or 645 cm^−1^), respectively.

The examination of the results of the calculated absorption spectra for the porphyrin molecules studied here (Table 5) revealed two important points that are: (1) the *meso*-substitutions of the porphyrin with the phenyl/sulfonatophenyl and the protonation of the N atoms at porphyrin core bring about significant red shift in the spectral position of the B-bands and Q-bands; (2) even though the ISC process between the B-band(s) and triplet states occurs for the all porphyrin derivatives, but, there would be an ISC process between the Q-bands (singlet) and triplet state(s) if both of the protonation and meso substitution of the porphyrin with the phenyl/sulfonatophenyl take place.

Figure 6 provides the electron densities in the HOMOs and LUMOs molecular orbitals and nonbonding atomic orbitals (n) involved in the electronic transitions (see Table 5). The plotted electron densities indicated that the HOMO (Hs) and LUMOs (Ls) are as a resluts of binding/antibinding (π/π*) and nonbinding (n) atomic orbitals beween the atoms within the macrocycle and phenhyl ring such as H: π(C_α_-C_m_-C_α_/C_β_-C_β_/C-C in phenyl)+n(N); (H-1) to (H-4): generates from the nobonding orbitals of oxygen atom, n(O); H-5: π(C_α_-C_β_); (H-6)-(H-8): π(C-C-C in phenyl)+ minor n(O and C_β_); L and (L+1): π*(C_α_-C_m_/C_β_-C_β_)+n*(N); L+2: π*(C_ϕ_-C_m_/C_β_-C_α_ ) and minor n*(C in phenyl); and (L+3)-(L+5): π*(C-C in phenyl)+n*(C(S) and C_ϕ_ in phenyl) for the H_2_TSPP. For the H_6_TSPP, H: π(C_α_-C_m_-C_α_/C_β_-C_β_)+n(N and C_ϕ_/C(S) in phenyl) H-1: π(C_α_-C_β_); (H-2)-(H-5): π(C-C-C in phenyl) + n(C_α_ and C_β_, minor); (H-6) and (H-7): π(C(S)-C/C-C_ϕ_ in phenyl and C_m_-C_α_-C_β_); L and (L+1): π*(C_α_-C_m_/C_β_-C_β_)+n*(N); L+2: π*(C_ϕ_-C_m_/C_β_-C_β_ and C-C/C-S in phenyl); (L+3)-(L+6): π*(C-C/C-S in phenyl) and n*( C_ϕ_)/n*(C_α_ and N, minor); and L+7: π*(C_β_-C_β_)+n*(N and C_α_).

### 2.5. Relaxed Potential Energy Surfaces (PESs) Scan of TSPP Molecule

**Figure 7:**
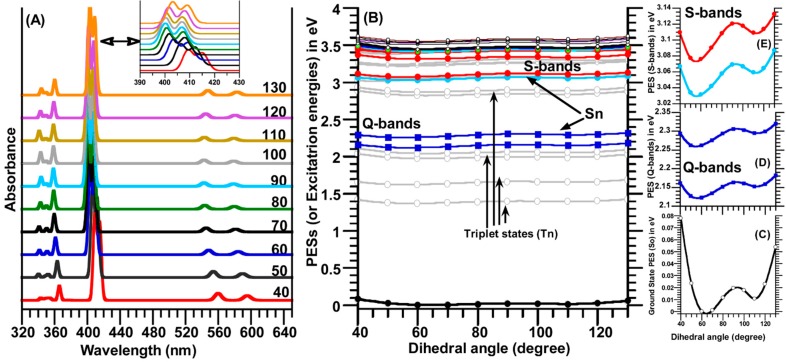
(**A**) The calculated electronic spectra (S_0_→S_n_, *n* = 1–24) and (**B**) relaxed potential energy surfaces (PESs) scan for the ground state S_0_ and upper states S_n_ and T_n_ (*n* = 1–24) of the TSPP molecule as function of the dihedral angle (C_α_-C_m_-C_ϕ_-C(ph)) rotation in the range of 40° to 130° with a step size of 10°; (**C**–**E**) display the change in the PES for the S_0_ (the ground state), Soret bands (B-bands) and Q-bands in low scale for a better view. It should be noted that only one of the four meso-sulfonatophenyl groups rotated around C_m_-C_ϕ_ bond. Note that the S-bands in Figure 7E indicates the Soret bands (or B-bands).

The ground state (S_0_) relaxed PES scan of the TSPP molecule was calculated in water (used as a solvent) by rotating one of four dihedral angles θ (C_α_-C_m_-C_ϕ_-C) in the region of 40° to 130° with a step size of 10°. As seen in Figure 7B,C, the calculated relaxed PES(S_0_) curve exhibited two minima at dihedral angles of ~66° and 110°.

These two minima on the relaxed PES curve of the ground state (S_0_), at 65° and 110°, correspond to the lowest ground state (with symmetry of C_2v_) and an energetically more stable local state (with symmetry of C_2_), respectively. The local minima (at 110° ) lies only 0.0132 eV (or 106 cm^−1^) above the lowest ground state at ~66°) for the TSPP molecule (Figure 7C). The highest potential energy barrier is only 0.0219 eV (177 cm^−1^) at the dihedral angle of 90° when the molecule going from the lowest ground state to this local state. This finding implies that the meso-sulfonatophenyl substitution groups can be easily rotated around C_m_-C_ϕ_ bond at room temperature since the thermal energy (kT) at 298 K is 207.2 cm^−1^. Therefore, the self-assembly or arrangement of the TSPP molecules in any environment might be very easily formed since the predicted potential energy barrier is low as much as 0.0132 eV/106 cm^−1^ (at the dihedral angle of 90°). It is should be point out that the calculated ground state relaxed PES scan was obtained for only one of four meso-sulfonatophenyl groups rotation within the TSPP molecule. If the relaxed PES scan is obtained for the rotations of all four meso-substitutional groups, there would be several different local minimum with a different potential energy barriers on the ground state PES. In this case, these small changes in the potential energy barrier distribution of the TSPP molecules may be used such as scanning nanocalorimetry measurement purpose or other electronic purposes that responding to very small changes in energy.

The PES scan of the singlet (S_n_) and triplet (T_n_) excited states were obtained by calculating the vertical electronic transitions S_0_→S_n_ and S_0_→T_n_ (*n* = 1–24) energies for each optimized ground state geometry of the TSPP molecule at the dihedral angle θ(C_α_-C_m_-C_ϕ_-C(ph)) and taking into account of the SCF energy corrections, using the following equation:

$$V_{n}(S_{n},\theta )=E(\theta )-E_{0}+E(S_{0}\;\to \;S_{n};\;\theta )$$

$$V_{n}(T_{n},\theta )=E(\theta )-E_{0}+E(S_{0}\;\to \;T_{n};\;\theta )$$

where E_0_ and E(θ) correspond to the calculated global (total SCF) energies at the lowest ground state and the relaxed potential energy at the dihedral angle θ(C_α_-C_m_-C_ϕ_-C_1_), respectively; and (S_0_→S_n_/S_0_→T_n_; θ) indicates the vertical electronic transition energy between S_0_ and excited energy levels S_0_/T_n_ at the rotated dihedral angle rotated θ. As seen in Figure 7C–E, change in the electronic energy levels of the singlet and triplet states as function of the dihedral angle rotation, PESs (for S_n_ and T_n_ states), are similar to change in the PES(S_0_) curve. The calculated dipole allowed electronic transitions at each rotated dihedral angle are given in Figure 7A. The results of the calculations displayed that the red shift in spectral position of Soret bands (B-band) increases with the increasing rotational dihedral angle in both right-handed and left-handed rotational direction around its equilibrium dihedral angle of ~66° in the ground state.

## 3. Theoretical and Experimental Techniques

### 3.1. Calculation Section

The ground state structures and vibrational modes of frequencies of the porphyrin and its derivatives in water used as solvent were calculated using density functional theory (DFT) with the B3LYP functional [33,34] with 6-311G(d,p) basis set [35]. The solvent effects were taken into account using the self-consistent reaction field (SCRF) calculations [36] with the conductor-like polarizable continuum model (CPCM) [37,38,39] with a dielectric constant of 78.39 for water, SCRF = (CPCM, Solvent = Water) as contained in the Gaussian 09 software package [40]. All compounds were optimized to minima on their ground state relaxed potential energy surfaces (PESs) that were verified by revealing no any imaginary frequency in their calculated vibrational spectra. In order to observe the deuteration effect on the vibrational spectra of the molecules studied here were calculated at same level of the DFT techniques. By using the time dependent DFT (TD-DFT), first twenty four singlet-singlet (S_0_→S_n_) and singlet-triplet (S_0_→T_n_. *n* = 1 to 24) vertical electronic transitions in water used as solvent were calculated at TD-B3LYP/6-31G(d,p) level of the theory. Finally, to explore the dependence of the potential energy of the ground state (S_0_) and excited states (S_n_ and T_n_) on the rotation of the C_m_-C_ϕ_ bond (or rotation of the dihedral angle θ(C_α_-C_m_-C_ϕ_-C(ph)), the relaxed potential energy surface (PES) scan was performed using the scan facility (keyword: “Opt = ModRedundant”) implemented within Gaussian09 [40]. The calculated ground state (S_0_) potential energies at each structure being optimized at the B3LYP/6-31G(d) level were plotted as a function of the rotated dihedral angle θ, ranging from 40° to 130° with a step size of 10°. The excited states (singlet (S_n_) and triplet (T_n_) states, *n* = 1 to 24) PES were obtained by calculations of the singlet-singlet (S_0_→S_n_) and singlet-triplet (S_0_→T_n_) electronic transition energies for each optimized structure at the rotated dihedral angle θ, including taking into account of the SCF energy correction (ΔE_SCF_ = E(θ) − E_0_) to the each electronic transition energy, where the E_0_ and E(θ) indicate the calculated global energies of the energetically most stable structure and the optimized structure at the dihedral angle θ, respectively.

Furthermore, vibrational mode descriptions were made on the basis of calculated nuclear displacements associated with measured vibrational frequencies, combined with visual inspection of the animated normal modes, to assess which bond and angle motions dominate the mode dynamics for the molecule. The DFT method was chosen because it is computationally less demanding than other approaches as regards inclusion of electron correlation. Moreover, in addition to its excellent accuracy and favorable computation expense ratio, the B3LYP calculation of Raman frequencies has shown its efficacy in our earlier studies, often proving itself the most reliable and preferable method for many molecular species of intermediate size, including carbon nanotubes (CNTs) and transition metals [41,42,43,44,45,46,47]. We would like to point out that the Raman, IR and electronic spectra of the molecules studied here were plotted using the GaussSum software [48]. The spectral resolutions were taken to be 4 cm^−1^ with the full width at half maximum (FWHM) of 12 cm^−1^ for the IR and Raman spectra (with the excitation energy 25,000 cm^−1^(400 nm)), and 60 cm^−1^ with the FWHM of 300 cm^−1^ for the electronic spectra. The calculated Raman and IR spectra were fitted to observed Raman and IR spectra using a fitting equation of ∆ν_sc_= 0.96∆ν_calc_ + 40.

### 3.2. Experimental Section

TSPP was purchased from Mid-Century Chemicals (Posen, IL, USA) and used without further purification. The protonated TSPP (H_2_TSPP) solutions were prepared by dissolving TSPP in acidic aqueous medium (HCl was used to adjusting the pH value at ~2 in the concentration of ~5 × 10^−5^ M). Raman spectra was excited under ambient temperature condition using a Coherent 899 dye-laser with stilbene as the laser dye, which was optically pumped by 514 nm radiation provided by a Coherent Innova 200 argon-ion laser. Exciting laser radiation was directed through an Olympus BH-2 microscope and focused by a ×50 microscopic objective as a diffraction-limited beam onto the sample. The sample was deposited as a thin film onto a smooth metallic silver surface, with the substrate serving to quench fluorescence of the porphyrin through the “heavy atom effect”. Raman scattering signals from the H_2_TSPP sample were collected through the same microscope in a back-reflection configuration, and dispersed by a 0.6-meter Spex 1877 spectrograph equipped with a 1200 groove/mm grating, a holographic notch filter, and a Spex Spectrum-1 CCD camera cooled to 140 K by liquid nitrogen. The pre-resonance Raman spectrum reported here, unless otherwise stated, result from accumulations of 30 scanned spectra, each with a 5 s integration period, and have been corrected by background subtraction. The uncertainty in Raman shifts with our instrumentation is estimated to be < ±2.0 cm^−1^.

## 4. Conclusions

The results of the calculated Raman and IR spectra of porphyrin and its derivatives in conjunction with the animation of the vibrational motions of the molecule showed if the peak(s) are only or mainly due to the vibrational motions of the *meso*-substitutions, H atoms covalently bound to the porphyrin core (and sulfonato groups (-SO_3_^−^)), the *meso*-substitutions, protonation and deuteration lead to a significant frequency shifts in these peaks positions. For instance, this peak (at about 1600 cm^−1^) in the unsubstituted porphyrin is caused by the macrocycle is red shifted to around 1564 cm^−1^ in TPP/TSPP (*meso*-phenyl/sulfonatophenyl substituted porphyrin) and to 1540–1520 cm^−1^ in H_2_TPP, H_2_TSPP and H_6_TSPP (protonated and *meso-*substituted porphyrin). The peaks observed and predicted at around 1600 cm^−1^ in the spectra of TPP/TSPP and their protonated structures are a result of the vibrational stretching of the phenyl rings only. In the low frequency region, the observed and predicted Raman peaks at about 200 and 334 cm^−1^ in the TPP are respectively shifted to 242 (red shift) and 314 cm^−1^ (blue shift) in the protonated TSPP (H_2_TSPP). The red-shift is due to sulfonato (SO_3_) groups and blue shift is due to the protonation of the porphyrin core. The deuteration of the porphyrin and derivatives exhibited a red shift in the peak positions such as two Raman bands at 1020 and 985 cm^−1^ in the spectrum of TSPP are blue shifted to 1036 and 1005 cm^−1^ in the protonated TSPP (H_2_TSPP), these peaks are red shifted to 1012 and 980 cm^−1^ in the spectrum of deuterated TSPP (D_2_TSPP). When the four N atoms at core are deuterated (D_4_TSPP), then, these two peaks are shifted from 1036 and 1005 cm^−1^ (in H_4_TSPP) to 1026 and 983 cm^−1^ in D_4_TSPP. The IR spectra of the compounds studied here showed similar trends.

The calculated electronic spectra of these molecules displayed that the Soret band(s) (B-band and Q-bands are red-shifted due to the meso-substitutions and protonation and all spectra indicated the existence of an internal conversion (CI) process from the B-band(s) to the Q-band(s). however, there is a possibility of the intersystem crossing process between the singlet (S_n_) and triplet (T_n_) excited states due to the potential energy surface touching and vibrational coupling, its occurrence depends on the excited states dynamics such as competition between the vibrational relaxation, the IC and florescence to the ground state and the ISC process, but its observed for the metalloid porphyrin metal complex systems. Finally, the calculated relaxed potential energy surface scan of the TSPP molecules as function of dihedral angle indicated the PES curve of the ground state (S_0_) of the ionic TSPP exhibited two minima at dihedral angle (C_α_-C_m_-C_ϕ_-C) of about 66° (corresponds to the lowest ground state) and 110° (the local minimum on the relaxed PES). The energy deference between these two minima is 0.0132 eV (or 106 cm^−1^) and the highest potential energy barrier is only 0.0219 eV (177 cm^−1^ when going from the lowest ground state to this local state; which is compatible with the thermal energy (kT) at 298 K is 207.2 cm^−1^.
